# Supplementary material for: DAXX drives de novo lipogenesis and contributes to tumorigenesis
Source: Nat Commun. 2023 Apr 12;14:1927. doi: 10.1038/s41467-023-37501-0 (PMC10097704; doi:10.1038/s41467-023-37501-0)
Supplement: Supplementary file 1 — Supplementary Information [file 41467_2023_37501_MOESM1_ESM.pdf]

Mahmud et al. Supplementary Figures

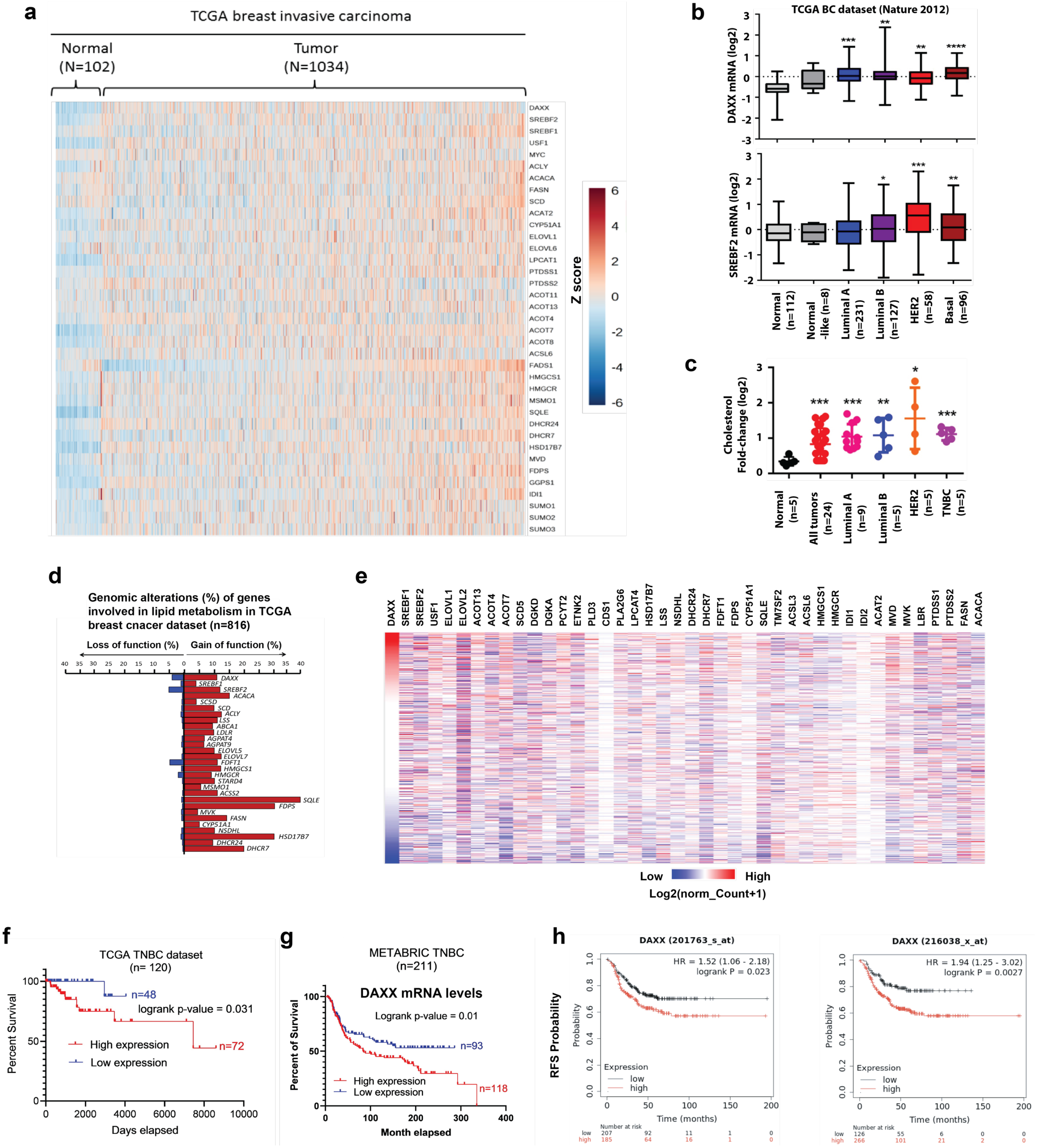

Fig. S1. **DAXX and lipogenic genes are highly expressed in breast cancer.**

(**a**) A gene expression heatmap of the indicated genes in normal breast tissues and breast cancer samples based on a TCGA breast cancer dataset (TCGA, Nature, 2012; PMID: 23000897).

(**b**) The mRNA expression levels for DAXX and SREBP2 in normal breast tissues and tumors of the indicated breast cancer subtypes based on an analysis of the TCGA breast cancer dataset as in a.

(**c**) Cholesterol levels in normal control and breast tumors of the indicated subtypes based on a published dataset (Tang X. et al., 2014; PMID: 25091696). Data are presented as mean values ± SEM.

(**d**) Genomic features of DAXX and the indicated genes in the lipid metabolism pathways. The percentages of breast tumor samples with the gain (mRNA upregulation or copy number gains), and the loss of gene functions (mRNA downregulation or copy number losses) are shown.

(**e**) A heatmap of relative mRNA levels of DAXX along with select genes in the lipid metabolism pathways. The red and blue groups refer to a high level (red) or low level (blue) of mRNA expression of the indicated genes according to combined expression scores in an individual tumor sample.

(**f**) A Kaplan-Meier plot of the correlation between gene expression levels of the selected genes in panel **e** (the red and blue groups) and patient survival time based on data of TNBC patients in the TCGA BC datasets.

(**g**) A Kaplan-Meier plot of TNBC patient survival based on DAXX mRNA levels in the METABRIC dataset.

(**h**) Kaplan-Meier plots of recurrence-free survival (RFS) for TNBC patients based on the DAXX mRNA expression levels using two distinct microarray probes. The "best cutoff" option was selected in the analysis. The plot is generated at kmplot.com.

The numbers of independent patient samples are denoted. P values: *: <0.05, **: <0.01, ***: <0.001 (panels **b** and **c**: unpaired two-tailed t-test).

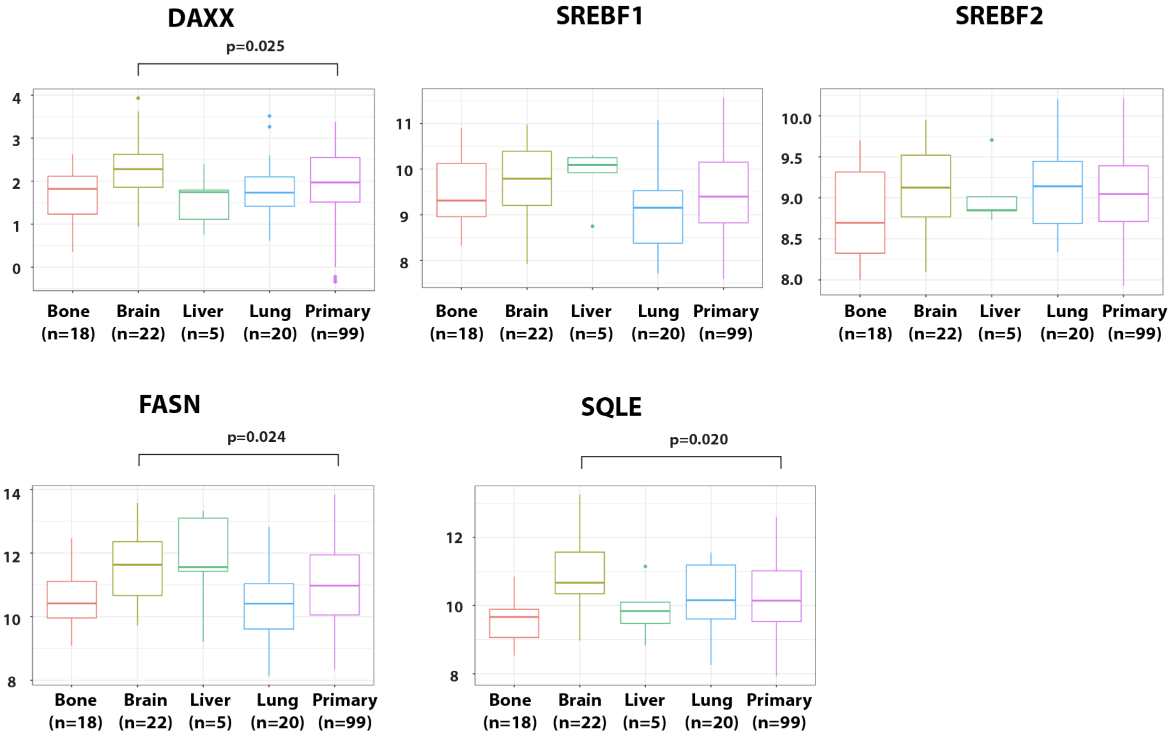

Fig. S2. DAXX and lipogenic genes are upregulated in BC brain metastases.

The mRNA levels of the indicated genes in the lipogenesis pathways were analyzed based on the microarray dataset GSE14020. The p-values were obtained via the two-sided student-t test. The box of the box plot represents the interquartile range (IQR) -- the range of the middle 50% of the data. The top of the box represents the upper quartile (75th percentile) of the data, and the bottom of the box represents the lower (25th percentile quartile) of the data. The center line represents the median value of the data set. The upper whisker is either the maximum value in the data set or the value that is 1.5 times the IQR above the upper quartile, whichever is lower. The lower whisker is either the minimum value in the data set or the value that is 1.5 times the IQR below the lower quartile, whichever is higher. If there are any values in the data set that fall outside the range between the lower whisker and the upper whisker, they will be displayed as individual points in the box plot.

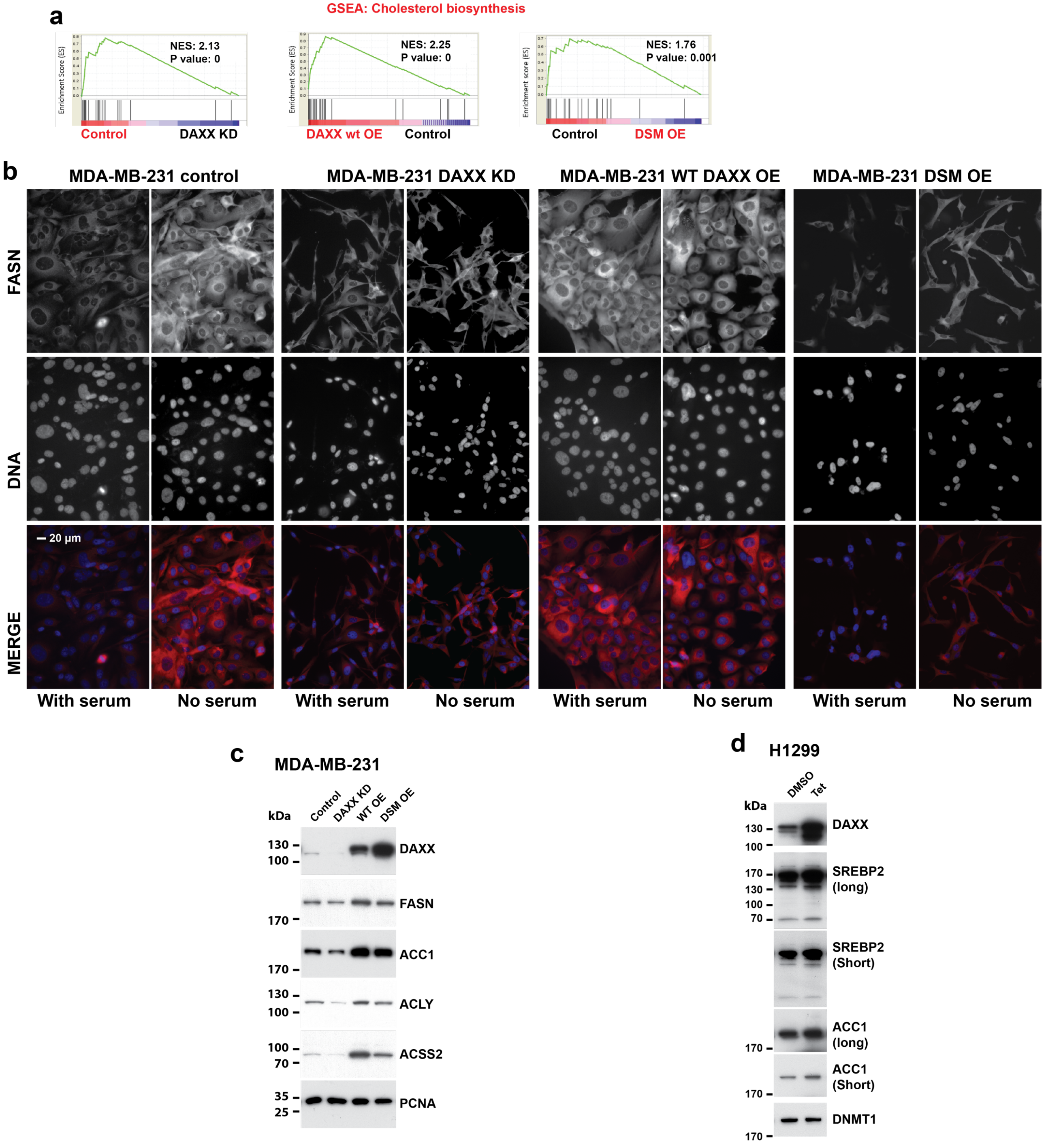

Fig. S3. **DAXX promotes lipogenic gene expression.**

(**a**) Gene set enrichment analyses (GSEA) of cholesterol biosynthesis genes downregulated due to DAXX KD or DSM OE (left and right) or upregulated due to DAXX WT OE in (center) MDA-MB-231 cells. The KEGG cholesterol biosynthesis geneset was used for the GSEA plots. GSEA defines an enrichment score (ES) via a weighted Kolmogorov-Smirnov statistic, and determines p-value of the ES using an empirical phenotype-based permutation test procedure.

(**b**) MDA-MB-231 cells stably transfected with a control vector (Control), a DAXX shRNA (KD), the wt DAXX, or the DSM mutant expression vector were cultured in the presence of serum or serum-starved for 24 hours. The cells were then fixed and stained with an anti-FASN polyclonal antibody (red) and counter stained with DAPI for visualizing nuclei (blue). The cells were imaged using a fluorescence microscope. All images were captured with the same duration of light exposure for the red or blue channel.

(**c**) Immunoblotting analysis of cell extracts of the four MDA-MB-231-derived cell lines with antibodies against the indicated proteins.

(**d**) Immunoblotting analysis of cell extracts of the H1299 cells expressing tetracycline (Tet)-inducible wt DAXX in the presence of control (DMSO) or Tet with antibodies against the indicated proteins.

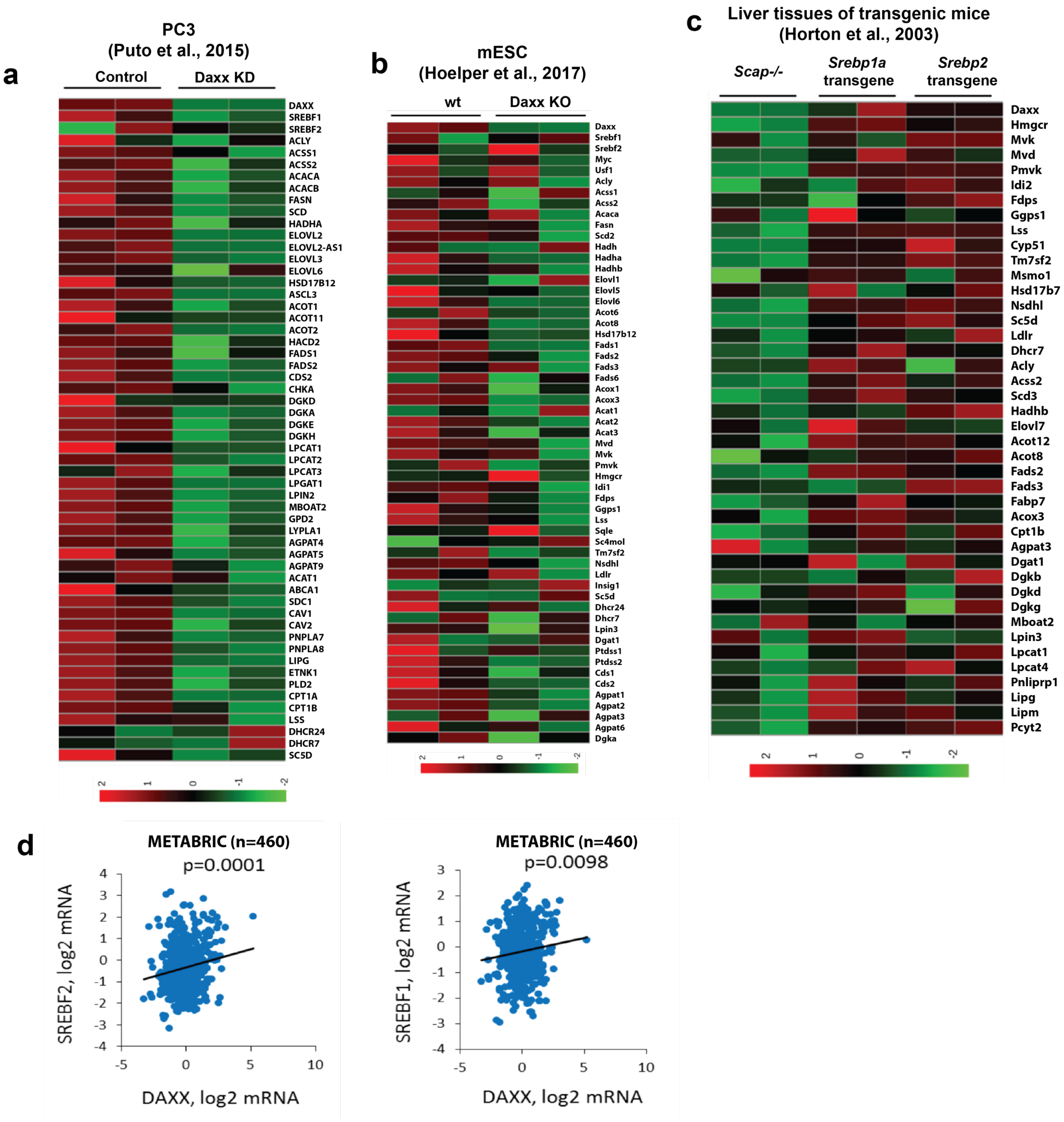

Fig. S4. **DAXX, SREBP1 and SREBP2 are key regulators for lipogenic gene expression.**

(**a**) A heatmap of the indicated lipogenic genes in control and DAXX KD cells of the human prostate cancer PC3 cell line (data from Puto et al., 2015, PMID: 25903140).

(**b**) A heatmap of the indicated lipogenic genes in wt and *Daxx* KO cells of the mouse embryonic stem cells (mESC; data from Hoelper et al., 2017, PMID: 29084956).

(**c**) A heatmap of the indicated lipogenic genes in liver tissues from mice with *Scap* deletion (Scap-/-), the nuclear form of Srebp1a or Srebp2 transgene (data from Horton et al., 2003, PMID: 14512514).

(**d**) Pearson correlation plots of the mRNA levels of *DAXX* vs. *SREBF1* or *SREBF2* based on the METABRIC breast cancer dataset. The p values were based on two-tailed unequal variance Student's t-test.

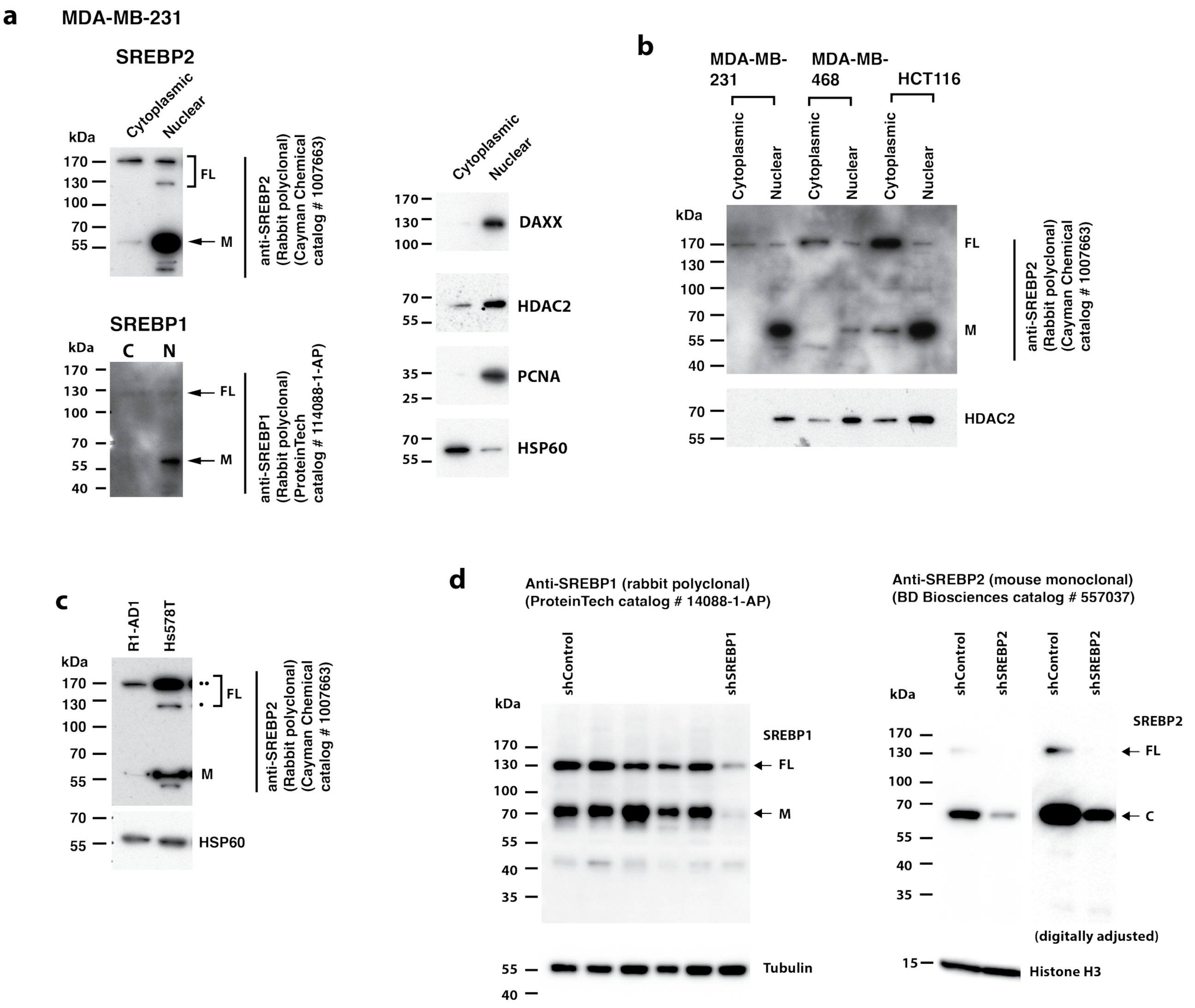

Fig. S5. **Validation of SREBP1 and SREBP2 antibodies used in this study.**

(**a** & **b**) MDA-MB-231, MDA-MB-468 (breast cancer) and HCT116 (colon cancer) cells were fractionated into cytoplasmic and nuclear fractions, which were analyzed in immunoblotting for SREBP2 (Cayman antibody) and SREBP1 (ProteinTech antibody). Note that the mature form (M) of SREBP2 (~55 kDa) and SREBP1 (between 55 and 70 kDa) were predominantly detected in the nuclear fraction. FL: the full-length (precursor) form of SREBP1/2.

(**c**) Whole cell lysates of the prostate cancer cell line R1-AD1 (a subline of CWR-R1) and breast cancer cell line Hs578T were subjected to immunoblotting using the Cayman antibody as above. Two larger bands of SREBP2 (likely the precursors, denoted with • and ••, respectively) were detected. FL: full-length (precursor) SREBP2. M: mature SREBP2.

(**d**) MDA-MB-231 cells with a control vector, an SREBP1 shRNA (TRCN0000020607), or an SREBP2 shRNA (TRCN0000020667) were subjected to immunoblotting with the indicated antibodies. C: cleaved C-terminal fragment of SREBP2.

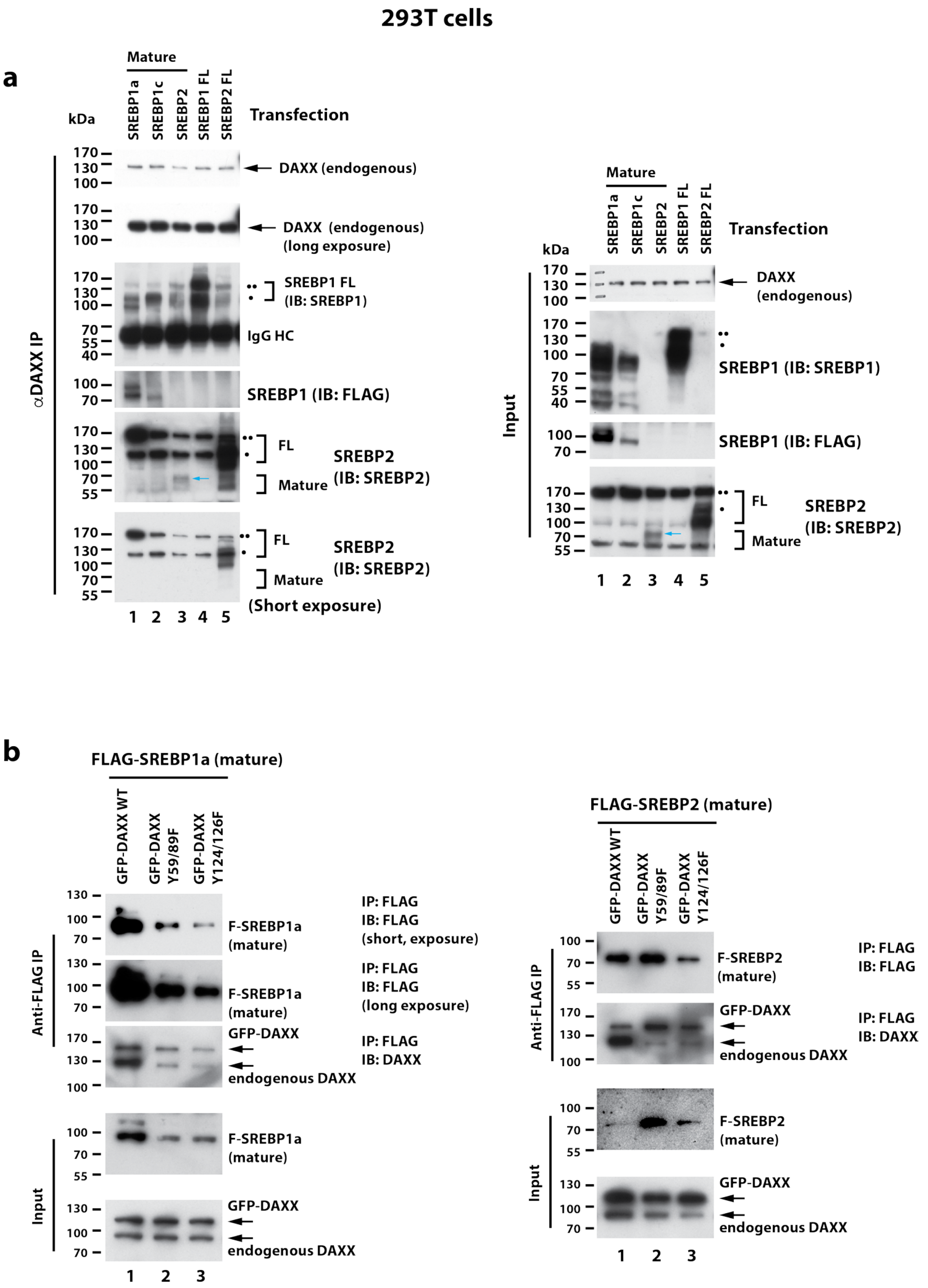

Fig. S6. **DAXX interacts with full-length and mature SREBP1 and SREBP2.**

(**a**) An expression vector for the mature form of SREBP1a (Addgene 26801), SREBP1c (Addgene 26802), or SREBP2 (Addgene 26807) as well as for the mouse full-length SREBP1 (Addgene 32017) or SREBP2 (Addgene 32018) was transfected to 293T cells. The total lysates of transfected cells were subjected to IP with the mouse anti-DAXX mAb (5G11) and immunoblotting with antibodies against DAXX (Bethyl Laboratories, A301-352A), SREBP1 (SC-13551, Santa Cruz Biotechnology), FLAG (Bethyl Laboratories, A190-102A-7) and SREBP2 (Cayman Chemical, 10007663). Note that two bands of FL SREBP1/2 were seen in the blots. In the

input samples (right panel), the upper band (~170 kDa, denoted with ••) was the major band in the absence of SREBP2 FL transfection (lane 1-4). In the DAXX immunoprecipitates, the upper band (~170 kDa) along with a band with an apparent molecular weight between 130 and 100 kDa, which is likely the canonical SREBP2 precursor (~125 kDa, denoted with •), was co-precipitated with DAXX (left panel). For SREBP1, both precursor bands (denoted with •• and •) were efficiently co-precipitated with DAXX. The transfected mature SREBP1a (lane 1), SREBP1c (lane 2), and FL SREBP1 (lane 4) were co-immunoprecipitated with DAXX. Similarly, the transfected mature (lane 3, denoted with a cyan arrow) and FL SREBP2 (lane 5) were co-immunoprecipitated with DAXX.

(**b**) Mature FLAG-SREBP1a (Addgene 26801) or SREBP2 (Addgene 26807) were co-transfected with the indicated GFP-DAXX constructs in 293T cells. The transfected cells were treated with 5 μM Z-VAD. At 24h after transfection, the total lysates of transfected cells were subjected to IP with the anti-FLAG mAb (M2, Millipore-Sigma F1804). Immunoblotting of the immunoprecipitates and the total cell extracts (input) was conducted using a rabbit anti-DAXX (Bethyl Laboratories, A301-352A) or anti-FLAG antibody (Cell Signaling Technology, #14793). The GFP-DAXX and endogenous DAXX are denoted.

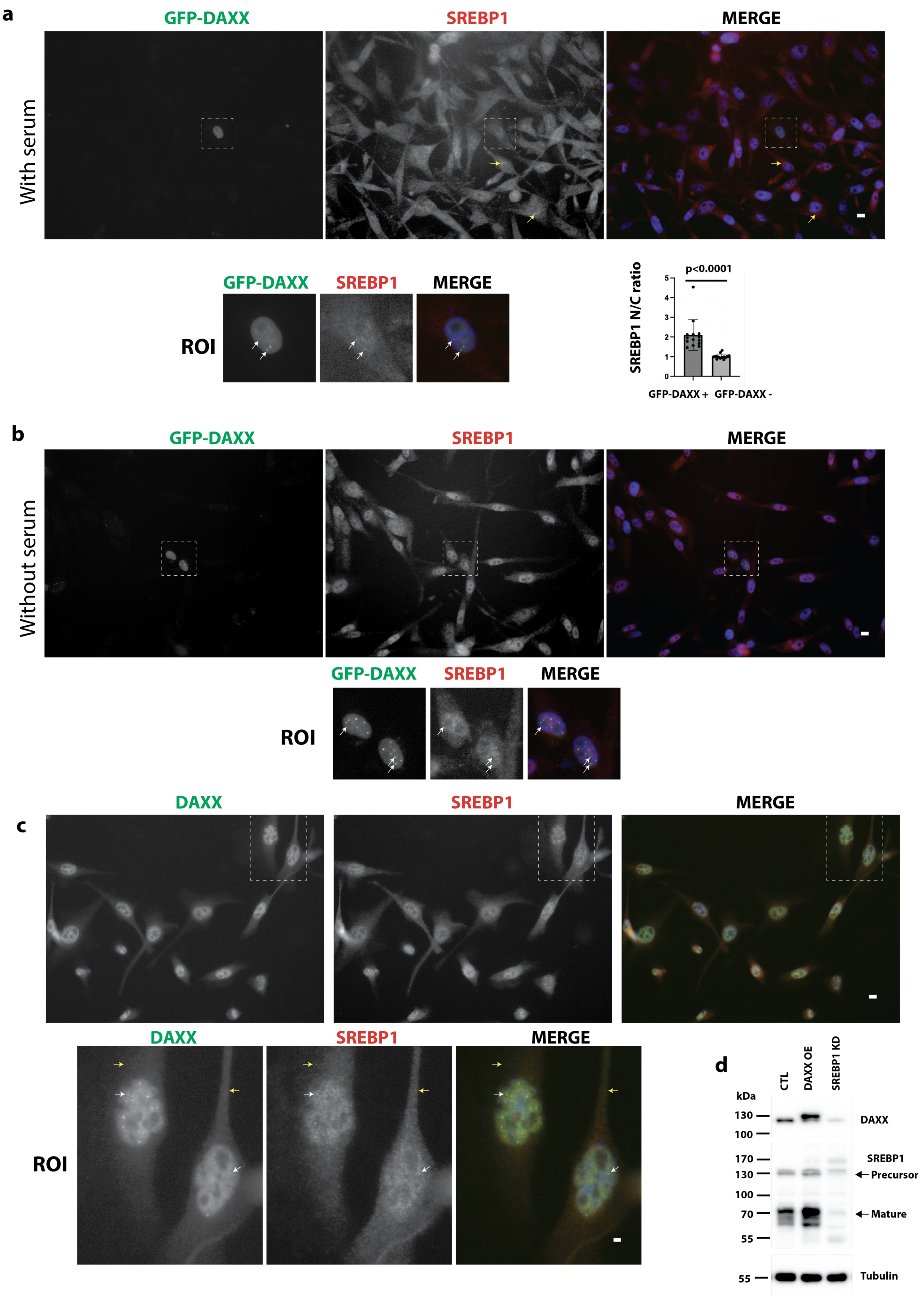
a
GFP-DAXX
SREBP1
MERGE
With serum
ROI
SREBP1 N/C ratio
p<0.0001
GFP-DAXX +
GFP-DAXX -
b
Without serum
c
DAXX
d
CTL
DAXX OE
SREBP1 KD
kDa
130
100
170
70
55
DAXX
SREBP1
Precursor
Mature
Tubulin

Fig. S7. **Colocalization of DAXX and SREBP1.**

(**a** and **b**) DAXX promotes SREBP1 nuclear translocation. MDA-MB-231 cells in medium with 10% serum (**a**) or without serum (**b**) were transfected with GFP-DAXX (WT). At 24 hour after transfection, cells were fixed and stained with an anti-SREBP1 antibody (ProteinTech catalog # 14088-1-AP). Colocalization of GFP-DAXX and SREBP1 were denoted with arrows in the indicated area of interest (ROI) in two representative transfected cells. In panel a, cells with high levels of perinuclear SREBP1 signals are pointed with yellow arrows. The nuclear to cytoplasmic signal intensity (N/C) ratio in cells expressing GFP-DAXX (GFP-DAXX+) vs. cells with no GFP-DAXX (GFP-DAXX-) in panel a were calculated based on relative gray levels measured by the Photoshop software. Data are presented as mean values ± SEM (n=14 different cells). The p value is based on unpaired two-tailed t-test. Microscopic scale bar: 10 µm.

(**c**) Colocalization of endogenous DAXX and SREBP1 in MDA-MB-231 cells in medium containing fetal bovine serum depleted of lipids. Colocalization of DAXX and SREBP1 in the nucleus (white arrows) and cytoplasm (yellow arrows) are denoted in the indicated ROI (scale bar: 10 µm in main image and 2 µm in ROI).

(**d**) DAXX overexpression increases levels of mature SREBP1. MDA-MB-231 cells with a control vector, WT DAXX cDNA, and SREBP1 shRNA were subjected to Western blotting with the indicated antibodies. The same anti-SREBP1 antibody (ProteinTech catalog # 14088-1-AP) was used for both IF and WB assays.

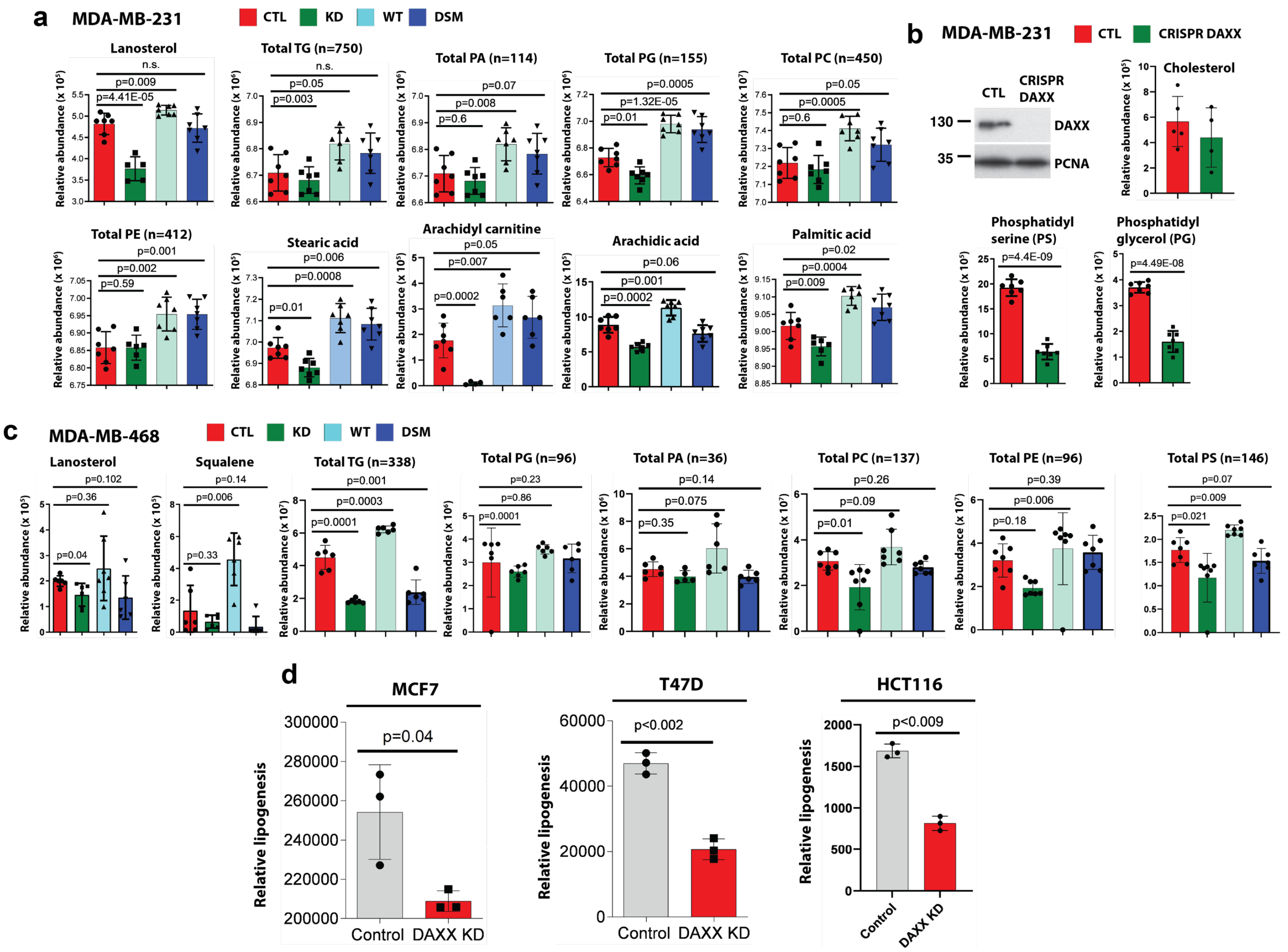

Fig. S8. **De novo lipogenesis correlates with levels of DAXX expression.**

(**a** and **c**) Mass spectrometry quantification of lipid species in cell lines (control: CTL, shDAXX: KD, WT DAXX OE: WT, or DSM OE: DSM) derived from the MDA-MB-231 (**a**) or MDA-MB-468 (**c**) cell line. Data are presented as mean values ± SEM (n=7). The p values were based on two-tailed unequal variance Student's t-test. PA: phosphatidic acid, PG: phosphatidyl glycerol, PC: phosphatidyl choline, PE: phosphatidyl ethanolamine, PS: phosphatidyl serine, TG: triacylglycerol.

(**b**) Mass spectrometry quantification of select lipid species in MDA-MB-231 cells with a control vector (CTL) and DAXX depletion by CRISPR/Cas9 (CRISPR DAXX). A validation immunoblot of DAXX depletion is shown. Mass spectrometry was done as above. Data presentation and statistics are as in **a**.

(**d**) *DAXX* knockdown reduces acetate-dependent lipogenesis in cancer cells. The luminal estrogen receptor-expressing BC cell lines MCF7 and T47D and the colon cancer cell line HCT116 with a control vector or a DAXX-specific shRNA were subject to de novo lipogenesis assay using [^{14}C] acetate labeling. The

radioactivity counts were normalized against total protein levels. Shown are average values (± SEM, n=3 biologically independent samples). The p values were based on two-tailed unequal variance Student's t-test.

a

de novo motif	Best match	Control	WT OE	DSM OE
		p value		
ATAAATTAAATT	TBP	1e-50	1e-60	-
GCTTCAGAGGGT	DCE	1e-28	1e-56	1e-20
GTGGATCTACCA	ZNF354C	1e-07	1e-53	1e-13
CCATGGAAGC	IRF4	1e-54	1e-49	1e-27
GGCTCAACACCA	BRCA1	-	1e-40	-
GACTTGGTGCCC	Hic1	1e-33	1e-38	-
TTTTTTATATAT	Mtf1	-	1e-38	-
TTCTCACAGCTC	Eomes	-	1e-36	-
ACAGAAGTCAAG	Rxra	-	1e-35	-
GCCCCTGTAG	Tcf	1e-34	1e-35	1e-22
GGGGCCAATGTA	Hnf4a	-	1e-33	1e-30
TGTAGACC	Zbtb12	-	1e-33	-
GCACGGCAGT	Zbtb3	-	1e-33	1e-94
GATCGCGAGC	Zbtb33	-	1e-23	1e-61

de novo motif	Best match	Control	WT OE	DSM OE
		p value		
CTGGCAGCAG	Tbx20 (T-box)	-	1e-33	-
GCATTGAGTGTC	Nkx2-5 (Homeobox)	-	1e-33	1e-21
TTCTGGGGTCTG	RUNX1	1e-29	1e-32	-
AGGTCCATGG	MAX	-	1e-32	-
ATGTGGGGTTGA	RUNX2	1e-32	1e-32	-
ACTCTGCGTGGG	Egr1	-	1e-22	1e-22
AATGTGGGGTCG	Glis2	-	1e-38	-
GGCTAACA	Bbx	-	1e-19	-
CTCCAGACGC	Smad4	-	1e-16	-
AGCAGCTG	FOXA1	1e-7	1e-15	1e-11
AGCAGCTG	Nf1 (CTF)	1e-10	1e-11	1e-02
AGCAGCTGCTGC	MyoD (bHLH)	1e-07	1e-11	1e-08
GTCATAAAAT	Cdx2	1e-10	1e-11	1e-03

de novo motif	Best match	Control	WT OE	DSM OE
		p value		
CCGGTCACGTGA	E-box (bHLH)	-	1e-10	-
GGCCACGTGG	c-Myc (bHLH)	1e-04	1e-11	1e-02
ATGGCGTGAA	Srebp1 (bHLH)	1e-03	1e-50	1e-25
GCACGTGCCT	Srebp2 (bHLH)	1e-02	1e-11	-
ATGGTAGATCCA	Nfkb1	1e-20	1e-63	-
GGTCACGTGA	USF1	1e-04	1e-10	-
GTCACGTGGT	USF2	-	1e-12	1e-08
AGGTCAATGACCTC	FXR (NR)	1e-04	1e-08	-

b

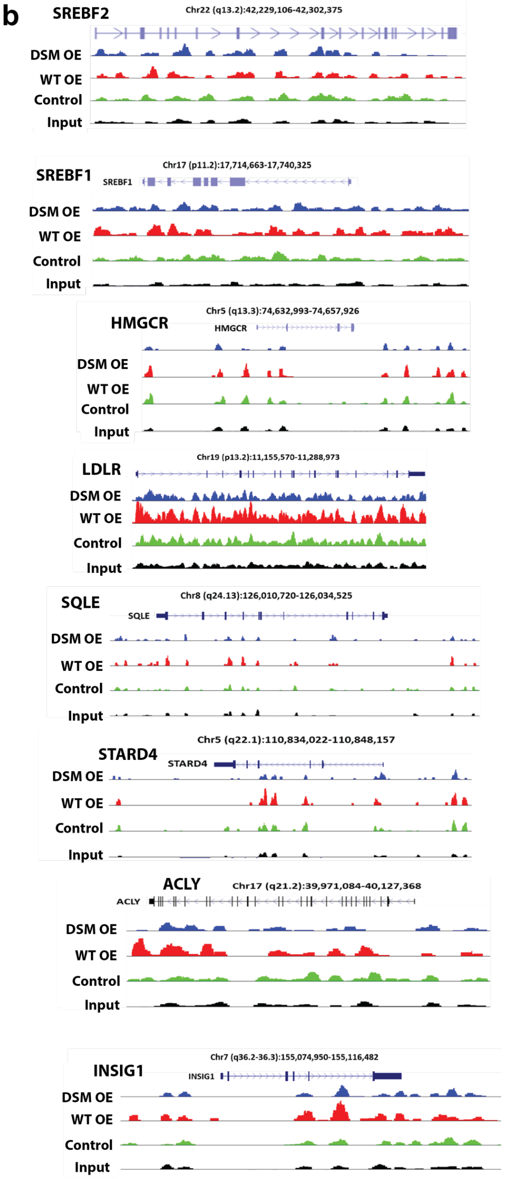

c

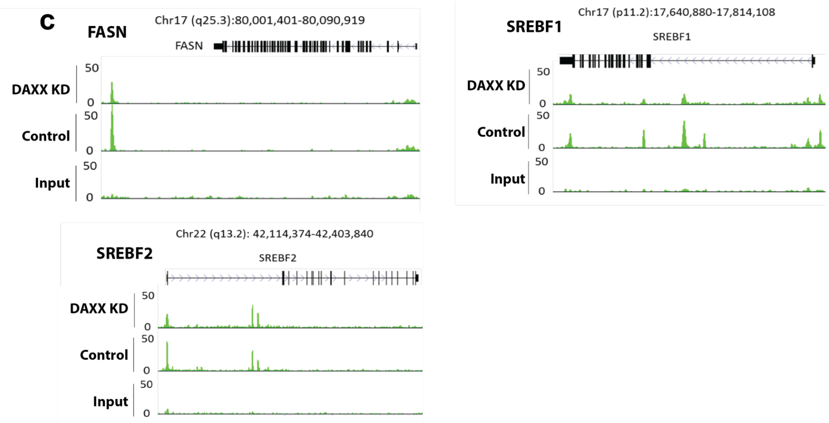

Fig. S9. **Chromatin-binding activity of DAXX.**

(**a**) De novo motifs associated with DAXX as revealed by ChIP-seq. DAXX ChIP-seq and motif analysis were done as in Fig. 4. Motifs enriched in MDA-MB-231-derived cells (control, wt OE and DSM OE) are shown. The HOMER software uses ZOOPS scoring (zero or one occurrence per sequence) coupled with the hypergeometric enrichment calculations (or binomial) to determine the p-value for motif enrichment.

(**b**) DAXX chromatin-binding profiles of the indicated individual lipogenic genes in MDA-MB-231-derived cells (control, wt OE and DSM OE) are depicted.

(**c**) DAXX chromatin-binding profiles of the indicated individual lipogenic genes in PC3-derived cells (control vs. KD; Puto et al., 2015, PMID: 25903140) are shown.

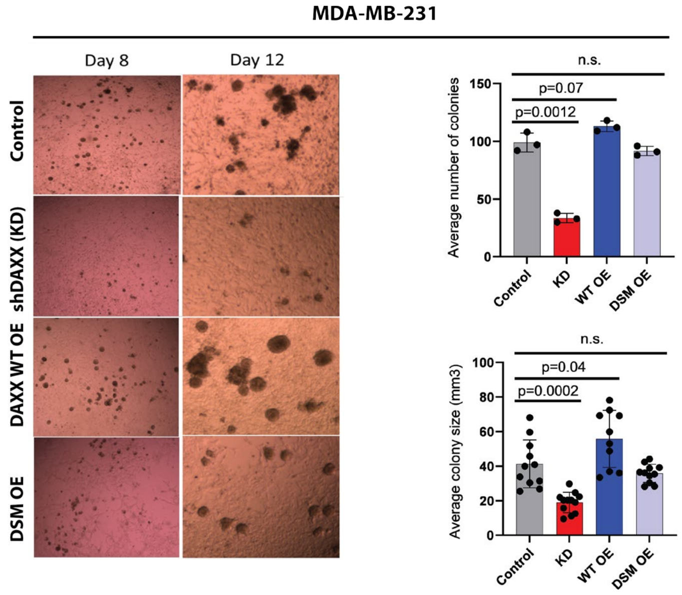

Fig. S10. DAXX promotes cell proliferation and 3D colony growth.

MDA-MB-231-derived cell lines were cultured in a suspension with Matrigel and complete DMEM medium. The 3D colonies of each line were imaged at the indicated time. Colony number and size were quantified at Day 12. Shown are average values (± SEM, n=3 biologically independent samples for colony number graph, and n=11 distinct colonies for the colony size quantification). The p values were based on two-tailed unequal variance Student's t-test.

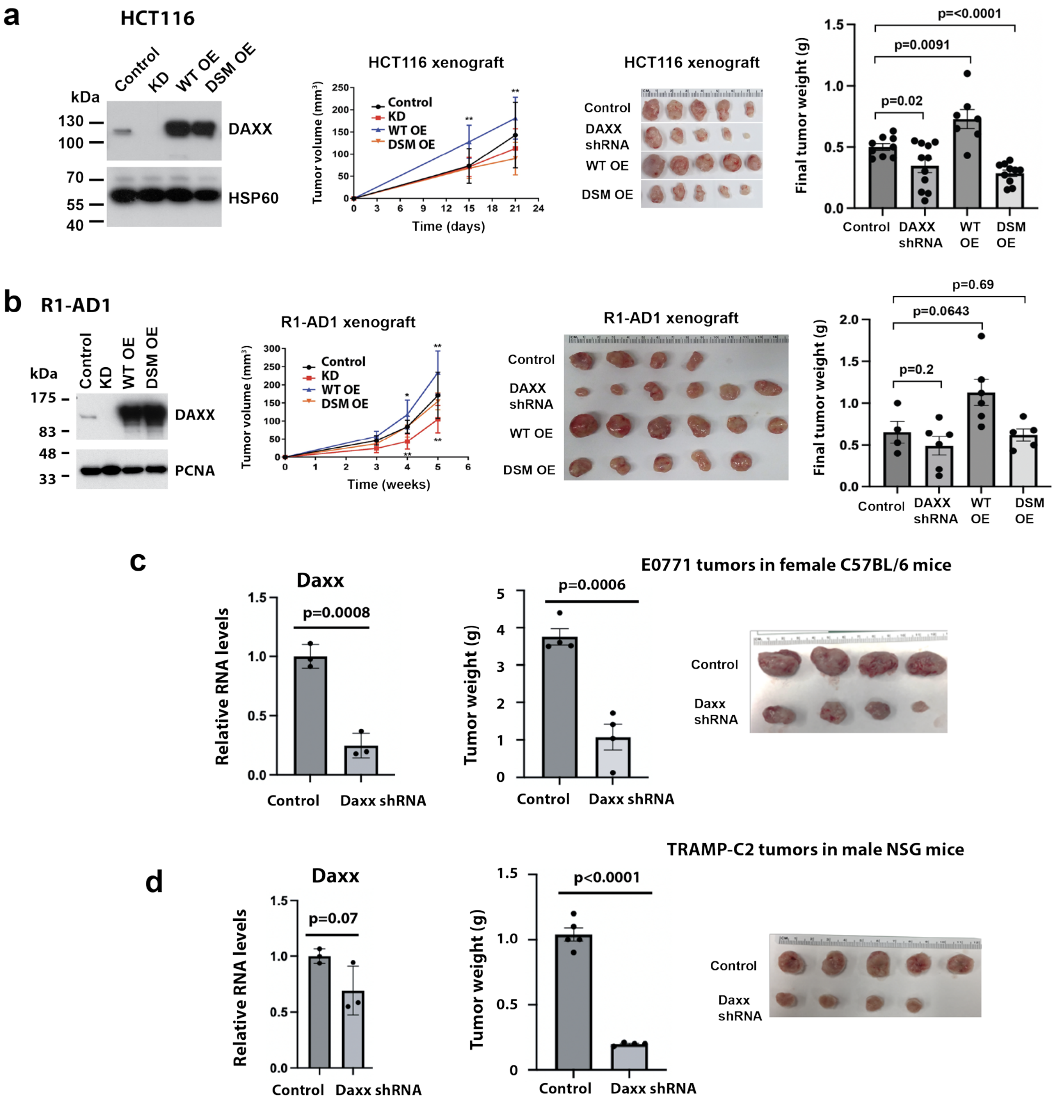

Fig. S11. **DAXX enhances in vivo tumor growth of different cancer types**.

(**a**) The DAXX protein levels in the four HCT116-derived cell line as assessed by immunoblotting (left). These cell lines were xenografted subcutaneously in a flank of NSG mice (both male and female, n=5-11 tumor-bearing mice). A tumor volume plot, representative images of dissected tumors and the final tumor weights are shown.

(**b**) The DAXX protein levels in cells derived from the prostate cancer cell line R1-AD1 with a control vector, DAXX KD, WT DAXX OE or the DSM OE are shown (left). These cells were mixed with Matrigel and transplanted to male NSG mice subcutaneously (n=4, 5, 6 tumor-bearing mice) as in (a). A tumor volume plot, images of dissected tumors and their weights are shown.

(**c**) The mouse breast cancer E0771 cells stably transduced with a control, or a mouse *Daxx* shRNA were subjected to RT-qPCR (left). The cells were engrafted into the mammary fat pads of female C57BL/6 mice (n=4). The final tumor weights are plotted (middle). The images of dissected tumors are shown (right).

(**d**) The mouse prostate cancer TRAMP-C2 cells stably transduced with a control, or a mouse *Daxx* shRNA were subjected to RT-qPCR (left). The cells were engrafted into male NSG mice subcutaneously (n=4 or 5 tumor-bearing mice) as in (b). The final tumor weights are plotted (middle). The images of dissected tumors are shown (right).

All data are presented as mean values ± SEM, and the p value are calculated based on unpaired two-tailed t-test.

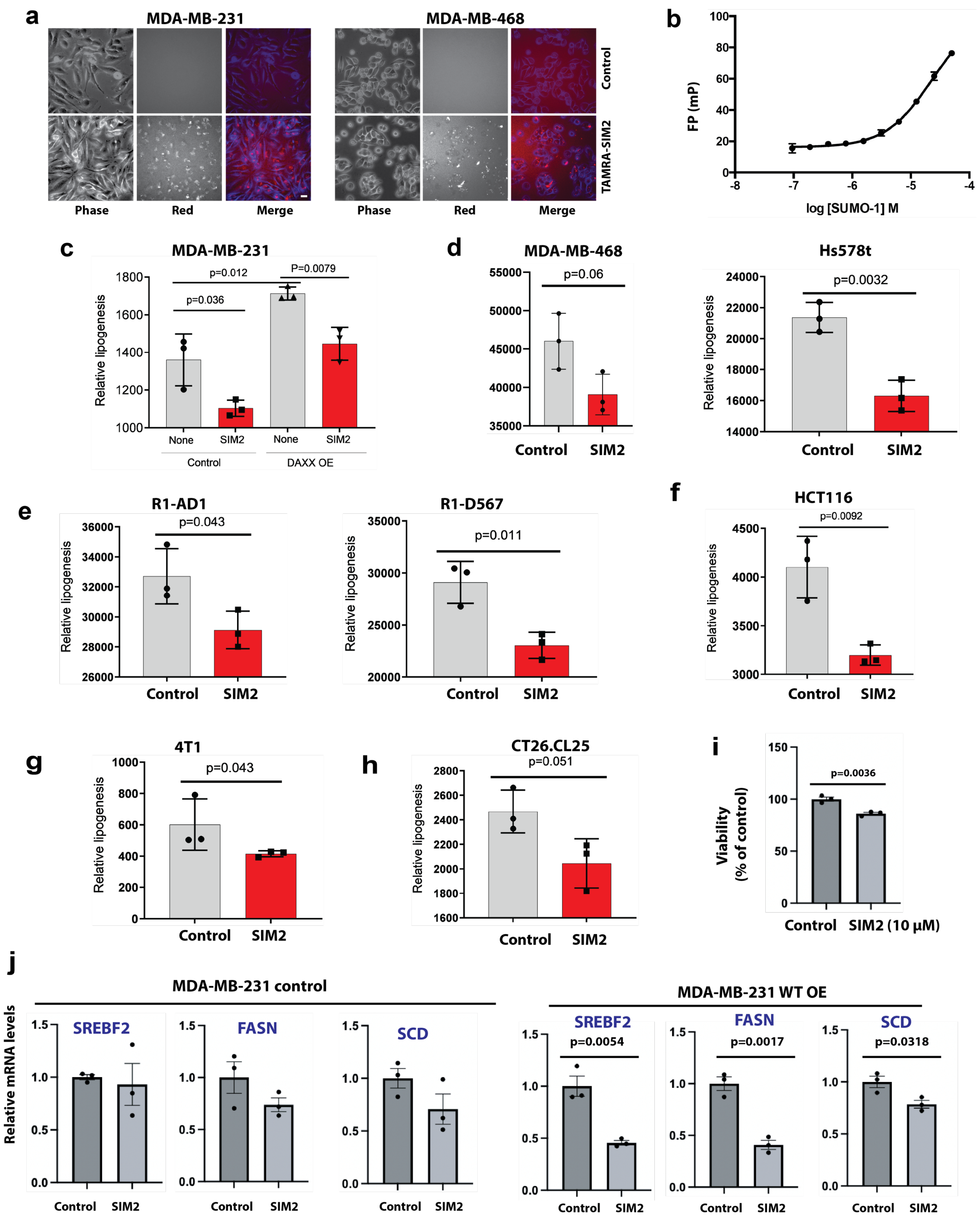

Fig. S12. **SIM2 peptide blocks lipogenesis**.

(**a**) The DAXX SIM2 peptide is rapidly internalized. MDA-MB-231 and MDA-MB-468 cells were exposed to TAMRA-SIM2 (1 μM) for 1 hour and visualized by microscopy. Microscopic scale bar: 20 μm.

(**b**) The SIM2 peptide binds to SUMO1. Fluorescence polarization assay was performed in the presence of TAMRA-SIM2 and increased concentrations of SUMO1.

(**c**-**h**) SIM2 blocks de novo lipogenesis in MDA-MB-231 control or DAXX OE (**c**), MDA-MB-468 (**d**), Hs578T (**e**), HCT116 (**f**), 4T1 (**g**), and CT26.CL25 (**h**). Serum-starved cells were untreated (control) or exposed to SIM2 (10 μM) and labeled with 1-[^{14}C] acetate. The levels of labeled lipids were quantified.

(**i**) SIM2 inhibits cell viability in vitro. DA-MB-231 cells were untreated (control) or treated with SIM2 (10 μM) for 48h (n=3 biologically independent samples). Viability was assessed using the PrestoBlue reagent.

(**j**) SIM2 inhibits lipogenic gene expression. MDA-MB-231 control and DAXX OE cell lines were untreated (control) or treated with SIM2 (10 μM) (n=3 biologically independent samples). At 24h after treatment, RNA was isolated and subject to qRT-PCR. RNA levels were normalized against that of *ACTB*. The p values shown are based on t-test.

All data are presented as mean values ± SEM, and the p value are calculated based on unpaired two-tailed t-test.

Source data for supplementary figures

Fig. S3

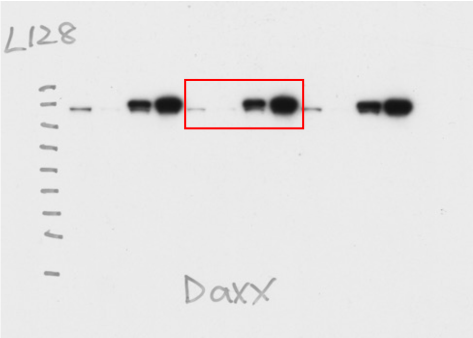

Supplementary Fig. S3c
DAXX WB

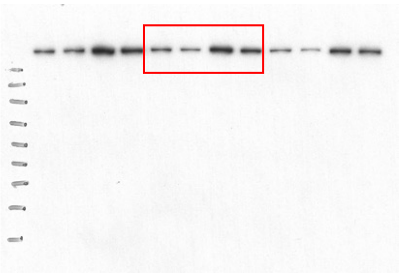

Supplementary Fig. S3c
FASN WB

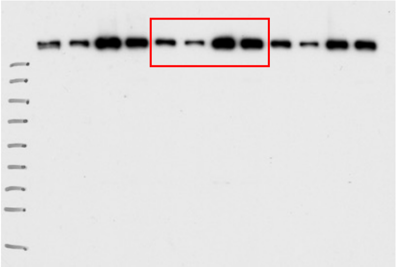

Supplementary Fig. S3c
ACC1 WB

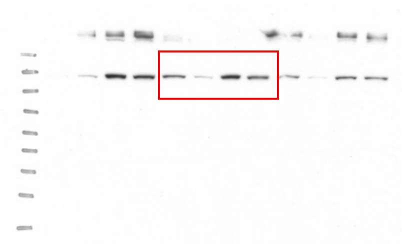

Supplementary Fig. S3c
ACLY WB

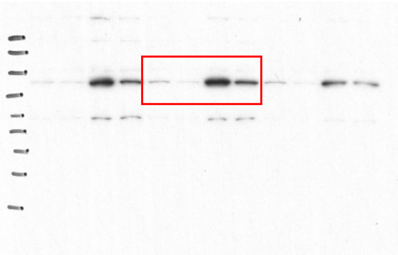

Supplementary Fig. S3c
ACSS2 WB

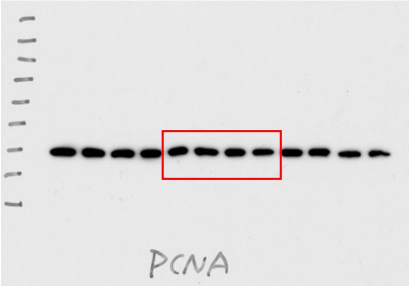

Supplementary Fig. S3c
PCNA WB

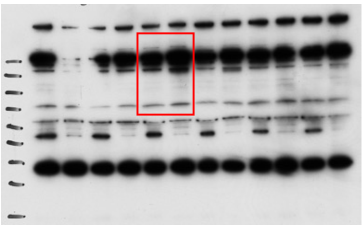

Supplementary Figure S3d, antibody: anti-SREBP2 (long)

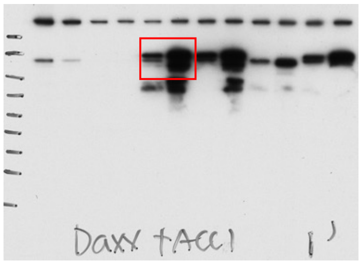

Supplementary Figure S3d, antibody: anti-DAXX

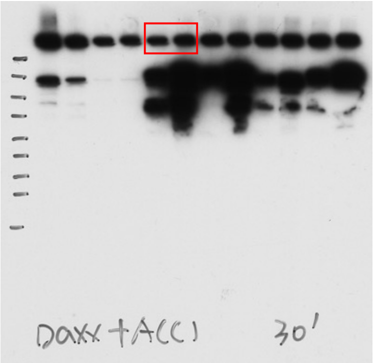

Supplementary Figure S3d, antibody: anti-ACC1 (long)

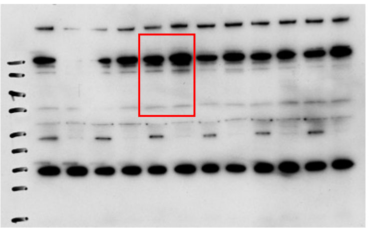

Supplementary Figure S3d, antibody: anti-SREBP2 (Short)

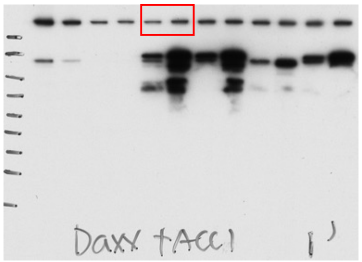

Supplementary Figure S3d, antibody: anti-ACC1 (short)

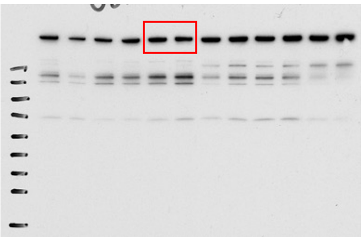

Supplementary Figure S3d, antibody: anti-DNMT1

Fig. S5

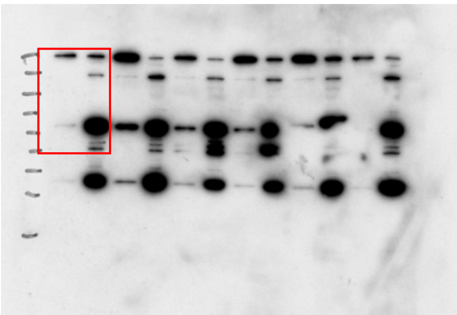

Supplementary Fig. S5b
SREBP2 WB

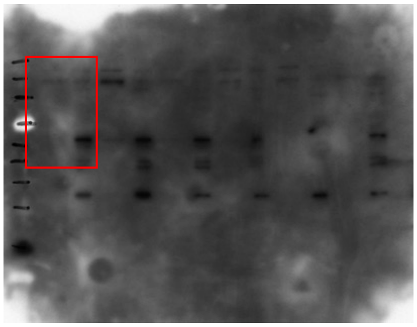

Supplementary Fig. S5b
SREBP1 WB

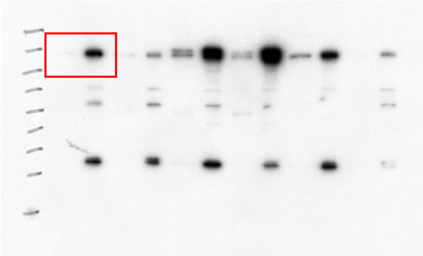

Supplementary Fig. S5b
DAXX WB

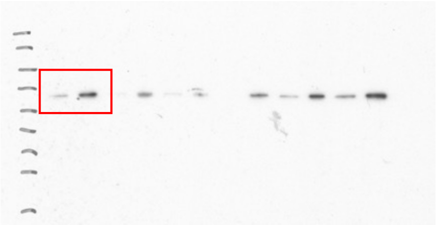

Supplementary Fig. S5b
HDAC2 WB

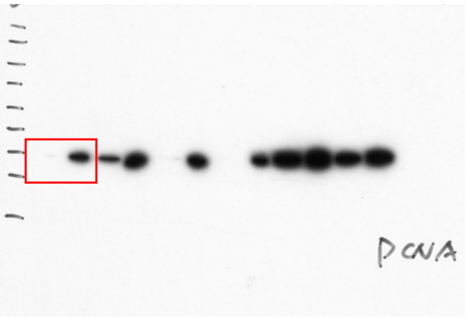

Supplementary Fig. S5b
PCNA WB

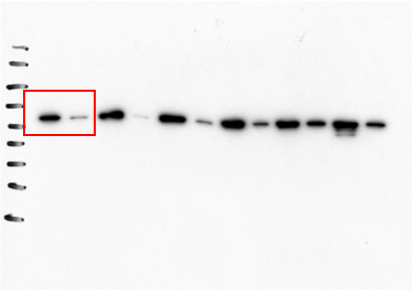

Supplementary Fig. S5b
HSP60 WB

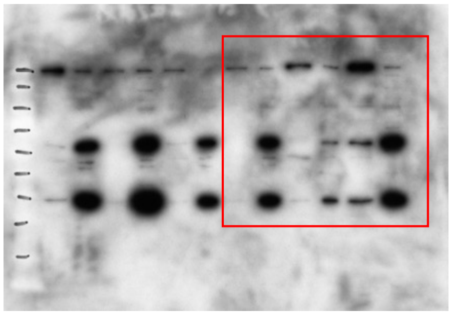

Supplementary Fig. S5c
SREBP2 WB

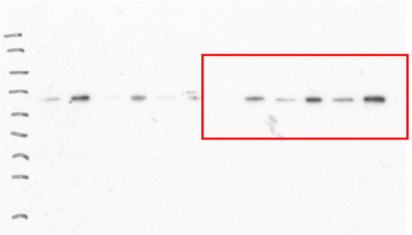

Supplementary Fig. S5c
HDAC2 WB

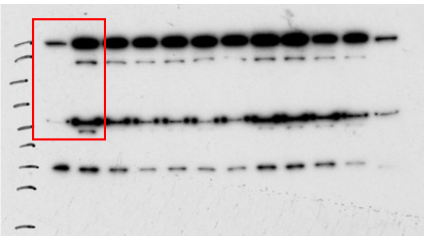

Supplementary Fig. S5d
SREBP2 WB

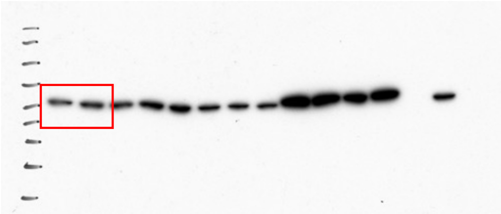

Supplementary Fig. S5d
HSP60 WB

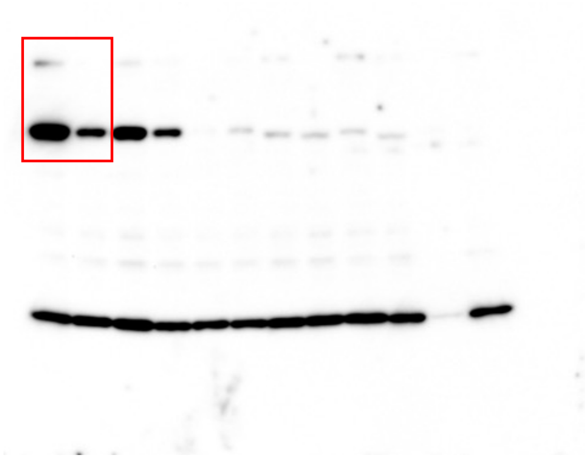

Supplementary Fig. S5e
SREBP2 WB

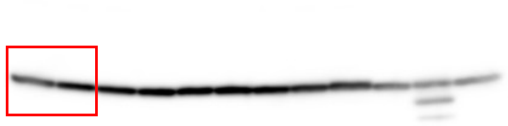

Supplementary Fig. S5e
Histone H3 WB

Fig. S6

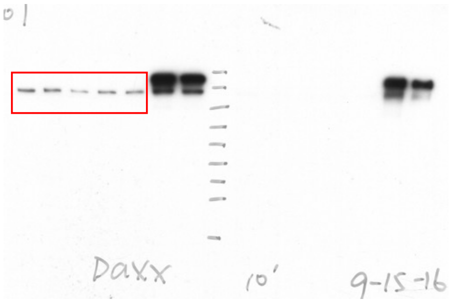

Supplementary Figure S6a Source file IP with anti-DAXX, WB with anti-DAXX #1

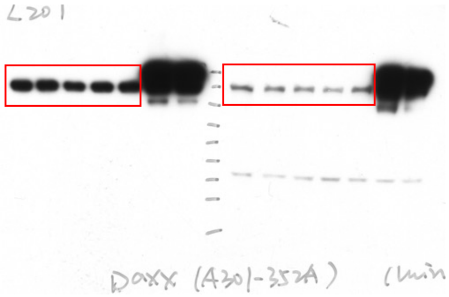

Supplementary Figure S6a Source file IP with anti-DAXX, WB with anti-DAXX #2

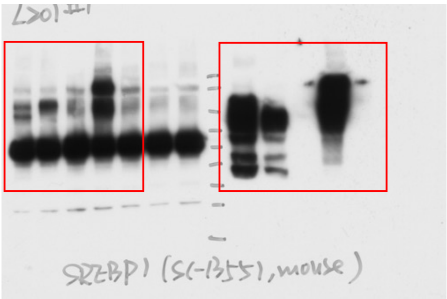

Supplementary Figure S6a Source file IP with anti-DAXX WB, with anti-SREBP1 #1

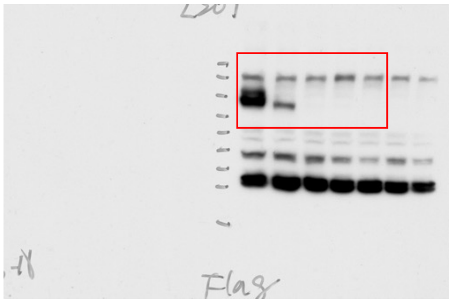

Supplementary Figure S6 Source file IP with anti-DAXX, WB with anti-FLAG #1

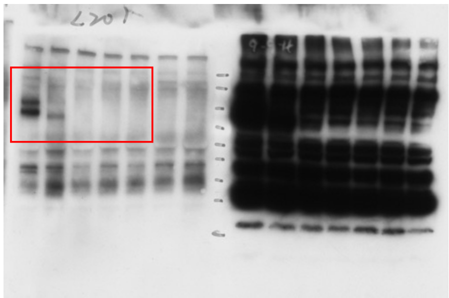

Supplementary Figure S6a Source file IP with anti-DAXX, WB with anti-FLAG #2

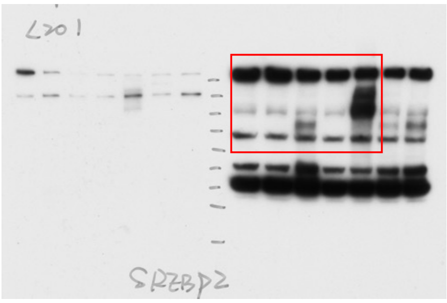

Supplementary Figure S6a Source file IP with anti-DAXX, WB with anti-SREBP2 #1

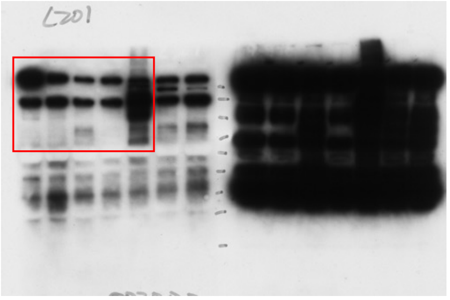

Supplementary Figure S6a Source file IP with anti-DAXX, WB with anti-SREBP2 #2

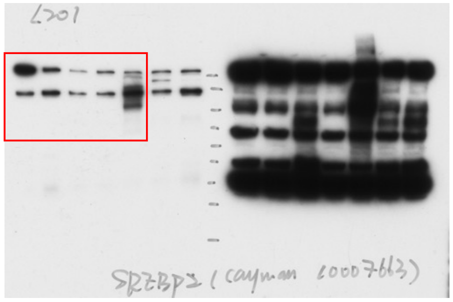

Supplementary Figure S6a Source file IP with anti-DAXX, WB with anti-SREBP2 #3

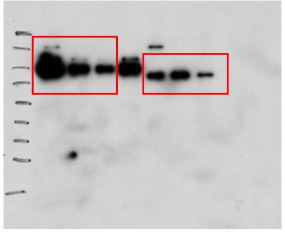

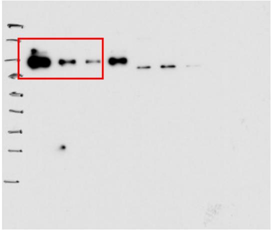

Supplementary Fig. S6b
IP, anti-FLAG (mouse), IB: anti-FLAG (rabbit). left: DAXX SREBP1a cotransfection, right: DAXX SREBP2 cotransfection

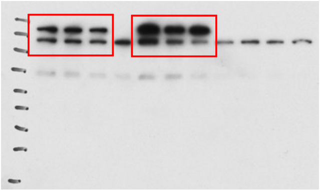

Supplementary Fig. S6b
Anti-DAXX, Input, left: DAXX SREBP1a cotransfection, right: DAXX SREBP2 cotransfection

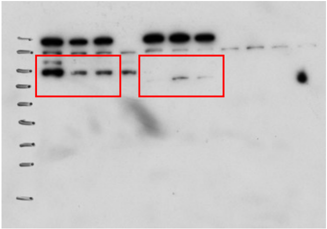

Supplementary Fig. S6b
Anti-FLAG, Input, left: DAXX SREBP1a cotransfection, right: DAXX SREBP2 cotransfection

Fig. S7a

SREBP1 signal intensity					
GFP-DAXX positive cells			GFP-DAXX negative cells		
Nuclear	Cytoplasmic	Ratio	Nuclear	Cytoplasmic	Ratio
19.15	9.58	2	23.89	23.28	1.03
18.03	10.15	1.78	16.47	17.34	0.95
20.26	10.81	1.87	14.8	17.04	0.87
14.39	7.39	1.95	19.8	23.7	0.84
21.2	13.51	1.57	9.6	9.8	0.98
21.96	12.03	1.83	10.79	11.78	0.92
10.58	3.76	2.81	17.17	16.83	1.02
12.83	6.02	2.13	23.03	23.17	0.99
10.11	4.66	2.17	19.5	19.12	1.02
6.95	4.56	1.52	17.28	18.02	0.96
8.88	4.16	2.13	15.39	14.65	1.05
12.43	2.74	4.54	17.61	17.78	0.99
18.62	11.12	1.67	17.7	13.4	1.32
15.13	10.34	1.46	19.8	16.4	1.21

Fig. S7d

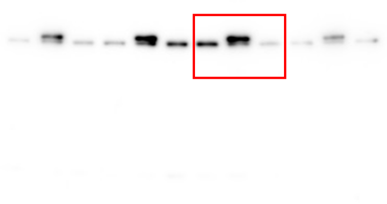

Supplementary Fig. S7d
DAXX WB

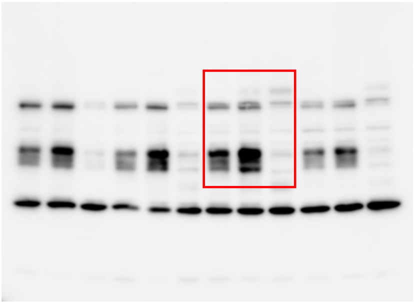

Supplementary Fig. S7d
SREBP1 WB

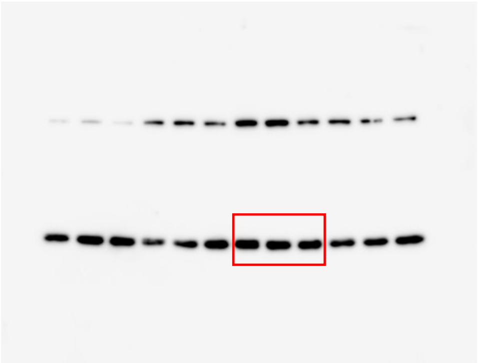

Supplementary Fig. S7d
Tubulin WB

Fig. S8a

Lanosterol

CTL	KD	WT	DSM
4.884289	3.477283	4.999014	4.718743
5.014591	3.49537	5.1463	4.175082
5.096876	4.138415	5.039907	4.478572
4.883511	3.852539	5.269287	5.027843
4.892372	3.859293	5.040681	4.79516
4.56685		5.250004	5.169352
4.388813		5.229513	4.669214

Triacylglycerol

CTL	KD	WT	DSM
6.919147	6.940491	7.106625	7.006951
7.060821	6.822748	7.123546	6.98037
6.988532	6.827865	6.997197	6.932897
6.964688	6.868216	7.053276	7.108016
6.987619	6.931155	6.954029	6.972337
6.968568	6.809076	7.025415	7.071377
6.99578	6.884089	7.028336	7.036405

Phosphatidic acid

CTL	KD	WT	DSM
6.802669	6.627239	6.897802	6.768112
6.634898	6.761504	6.8375	6.788928
6.679337	6.701138	6.750731	6.66315
6.698485	6.711327	6.867814	6.887291
6.785836	6.642573	6.724321	6.712002
6.628045	6.697837	6.821714	6.831597
6.732336	6.627887	6.83644	6.833562

Phosphatidylglycerol

CTL	KD	WT	DSM
6.791011	6.486238	7.057772	6.910208
6.697066	6.695823	7.053418	6.881722
6.67329	6.612311	6.922781	6.784304
6.717786	6.56368	7.01557	7.064218
6.826079	6.617853	6.890624	6.911739
6.631488	6.585807	6.941204	7.04321
6.758273	6.600229	6.975326	6.967124

Phosphatidylcholine

CTL	KD	WT	DSM
7.319544	7.08346	7.484645	7.333726
7.162153	7.328999	7.491064	7.313579
7.134125	7.159482	7.366689	7.21208
7.224834	7.196316	7.445054	7.439552
7.330983	7.212648	7.299091	7.189403
7.117484	7.12381	7.397496	7.403987
7.243558	7.173867	7.394791	7.35198

Phosphatidylethanolamine

CTL	KD	WT	DSM
6.919237	6.804288	6.996646	6.956099
6.816201		7.017022	6.938328
6.839927	6.864714	6.919529	6.890896
6.867038	6.867719	6.980483	7.003681
6.911375	6.837055	6.886976	6.912333
6.79653	6.911923	6.911005	7.00396
6.854152	6.861526	6.968222	6.970941

Stearic acid

CTL	KD	WT	DSM
6.927022	6.948816	7.20121	7.073445
7.066259	6.820674	7.128927	7.056027
6.944186	6.910554	6.998587	6.969096
6.976396	6.873973	7.171125	7.190144
6.991001	6.901428	7.065686	7.038216
6.924906	6.850029	7.093967	7.154528
6.979655	6.857291	7.126029	7.103522

Arachidyl carnitine

CTL	KD	WT	DSM
1.737155	0	4.320515	3.465351
1.419638	0	3.465351	1.549654
1.95003	0	3.69059	3.69059
0.633494	0.045497	2.001751	2.001751
2.73305	0.128754	2.669087	2.669087
1.5328	0.169072	2.666357	2.666357
2.352422	0.064435	0	0

Arachidic acid

Palmitic acid

CTL	KD	WT	DSM
8.401523	6.540875	12.13889	8.134176
8.391152	4.933609	9.946217	7.813541
10.18941	5.65078	12.32376	6.707029
8.225431	5.050683	11.5359	5.661248
10.13953	6.065301	9.590395	8.363223
7.110227	5.72501	12.11974	9.348459
9.58215	5.979653	11.57926	7.276228

CTL	KD	WT	DSM
9.017033	8.946452	9.130334	9.103804
8.977266	8.96708	9.123852	9.089905
9.033424	8.962843	9.071882	9.029384
9.029384	8.974051	9.11059	9.120574
9.075547	8.908485	9.060698	9.021189
8.956168	8.984527	9.120574	9.049218
9.025306		9.100371	9.075547

Fig. S8b

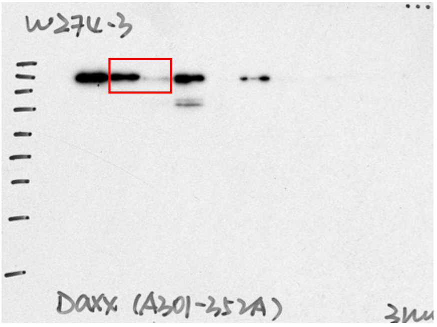

Supplementary Figure S8b, antibody: anti-DAXX

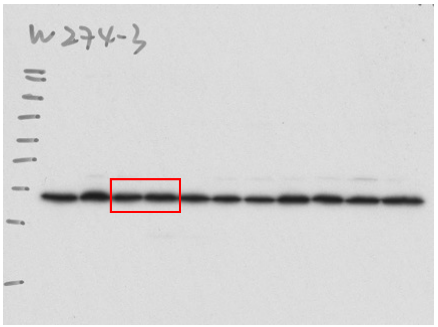

Supplementary Figure S8b, antibody: anti-PCNA

Cholesterol	
CTL	CRISPR DAXX
6.680909	6.819861
4.728359	5.773139
8.540402	3.344235
4.883482	1.653195
3.4675	0

Phosphatidylserine	
CTL	CRISPR DAXX
19.5483	7.486438
21.65032	8.939382
20.39473	6.208562
16.33793	6.068574
19.62125	5.850105
18.72226	6.358058
18.36056	3.84596

Phosphatidylglycerol

CTL	CRISPR DAXX
3.424461	1.875168
3.978326	2.172949
3.597132	1.873698
3.473458	1.391383
3.829011	0.987634
3.75884	1.265469
3.868598	1.635146

Fig. S8c

Lanosterol

CTL	KD	WT	DSM
2.049699	1.510061	3.641649	1.295364
2.004213	0.837221	2.38422	1.867419
2.002544	1.883557	2.016288	0.860447
2.32091	1.746001	4.687437	0.608434
1.92962	0.976746	2.12511	0.683962
1.666083	1.79312	0.987683	2.801251
		1.61874	

Squalene

CTL	KD	WT	DSM
2.590361	0.30366	4.518712	1.645767
0.12466	0.183807	5.973169	0.085546
0.615997	1.116284	5.832893	0.089244
0.621017	0.403317	1.730174	0.074838
0.155498	1.029602	3.694845	0.090544
3.983076	0.888233	5.629663	0.075226
0.657866	1.19445	0	0.328608

Triacylglycerol

CTL	KD	WT	DSM
4.857631	1.759485	6.322505	3.842518
4.663224	2.081484	6.061648	2.352443
3.714579	1.729043	6.527173	2.273472
4.745706	1.89036	5.940286	1.752111
5.533442	1.742656	6.197564	2.104904
3.556071	1.81183	6.309364	1.950934
4.729348	2.186061	6.554528	3.738362

Phosphatidylglycerol

CTL	KD	WT	DSM
3.770794	2.899373	3.666428	2.691469
3.793906	2.232582	3.487897	2.209224
3.180968	2.826259	3.251965	3.79892
3.791137	2.576684	3.473245	3.135543
3.398699	2.605073	3.848557	3.654967
0	2.439677	3.644446	3.519538
0	0	0	3.567838

Phosphatidic acid

CTL	KD	WT	DSM
3.809085	4.639331	4.289647	3.987694
4.626244	4.192731	7.772077	3.309928
4.174978	3.608111	4.575165	4.208428
4.310987	3.723213	4.707979	3.705283
4.851301	3.731217	8.460208	4.718381
5.325429	4.177271	6.355727	3.812573
4.735137	0	5.007285	4.683363

Phosphatidylcholine

CTL	KD	WT	DSM
3.591699	1.173427	3.543578	2.559169
3.448837	2.192341	3.106621	3.237111
2.545365	2.679141	5.08699	2.932038
2.823777	2.229205	4.421477	2.524804
2.899872	2.560254	2.938618	3.010147
2.785413	2.616591	3.25255	2.728161
3.436009		3.430559	2.539499

Phosphatidylethanolamine			
CTL	KD	WT	DSM
3.599705	2.42537	4.657222	2.696124
2.749096	1.665543	4.347922	4.77324
1.944144	1.687895	4.089847	4.123978
2.783363	1.618602	4.3947	2.987202
4.243992	1.690336	4.253424	3.970375
3.754602	2.225941	4.473294	3.734938
3.335752	2.225941	0	2.742842

Phosphatidylserine			
CTL	KD	WT	DSM
1.931574	1.220466	2.284384	1.665622
1.931959	1.375766	2.227737	1.954183
1.463298	1.330396	2.089541	1.49261
1.613048	1.408254	2.079812	1.112392
1.803861	1.429166	2.212679	1.446264
2.171236	1.429166	2.110444	1.389717
1.449435	0	2.377133	1.668481

Fig. S10

Colony number

Control	KD	WT OE	DSM OE
92	33	118	88
108	38	109	91
97	30	112	96

Colony size

Control	KD	WT OE	DSM OE
30.48389	29.87129	69.24038	36.24627
33.89164	22.036	59.98962	34.57566
45.77297	22.47898	36.14411	38.83966
59.68065	20.41	78.21689	39.28139
51.83378	20.41	48.77118	36.61838
68.16404	15.7783	53.12542	30.48389
26.87407	11.10158	33.56277	42.41906
25.55775	24.82388	72.49253	44.2116
40.0273	19.09987	69.31154	35.42081
31.55661	20.41	36.95341	28.43391
40.94059	12.56		28.26
	9.42		

Fig. S11

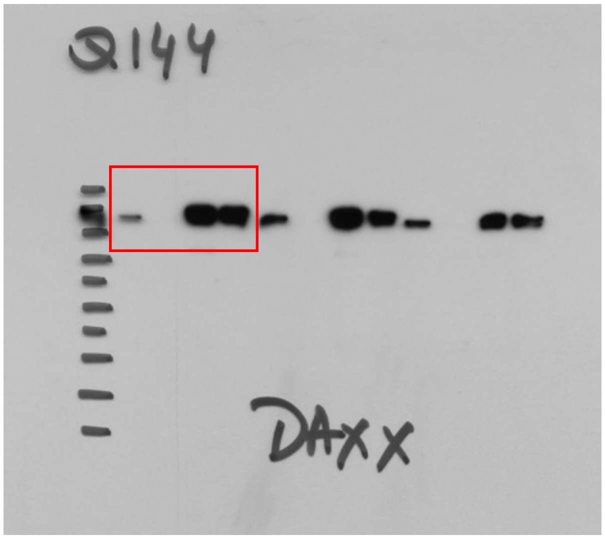

Fig. S11a DAXX WB

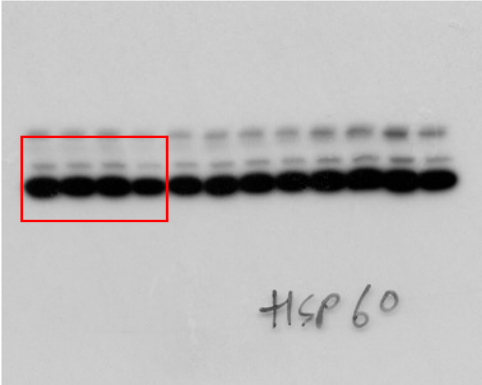

Fig. S11a HSP60 WB

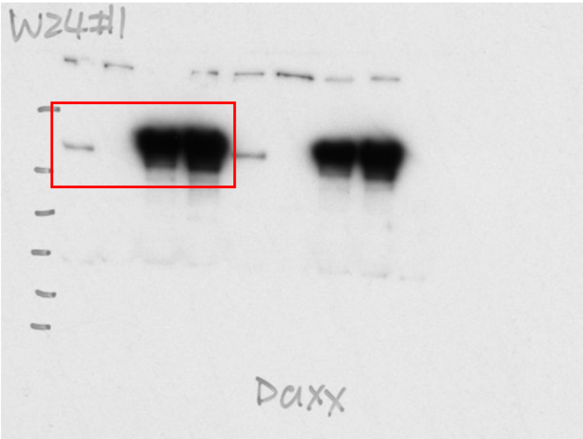

Fig. S11b DAXX WB

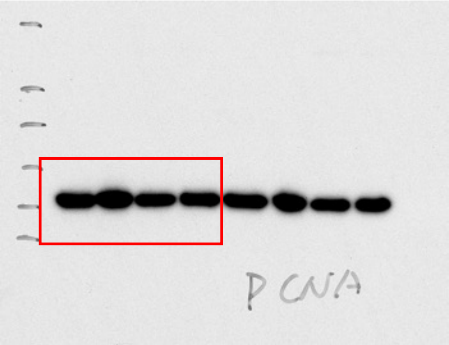

Fig. S11b PCNA WB

Fig. S11a

Tumor volumes (mm³, HCT116 xenografts)							
HCT116 CTL-Day15	HCT116 CTL-Day21	HCT116 KD-Day15	HCT116 KD-Day21	HCT116 WT OE-Day15	HCT116 WT OE-Day21	HCT116 DSM OE-Day15	HCT116 DSM OE-Day21
65.52	141.8	88.6375	127.204	197.2	269.988	54	89.824
55.3548	121.6035	30.8415	53.16	92.43	182	88	58.8
37.6	77	64.4233	81.18	111.36	143	48.4	58.4
108.9927	207.24	86.2745	168	132		48	102
133.777	250.9974	81.438	96			102	74.675

Tumor weight			
Control	KD	WT OE	DSM OE
0.53	0.54	1.1	0.39
0.58	0.55	0.76	0.31
0.47	0.3	0.73	0.22
0.42	0.23	0.83	0.16
0.63	0.12	0.43	0.15
0.57	0.06	0.651	0.31
0.5	0.13	0.605	0.321
0.41	0.412		0.36
0.4	0.411		0.35
	0.51		0.271
	0.532		

Fig. S11b

Tumor volumes (mm³, R1-AD1 xenografts)					
R1AD1-CTL W3	R1AD1-CTL W4	R1AD1-CTL W5	R1AD1-KD W3	R1AD1-KD W4	R1AD1-KD W5
48.36	74.6636	188.839	10.85	26	126.5264
36.4	72.52	140.7	14	21.115	128.7648
50.22	93.17	251.064	20	27.12	97.35
45.05	86.94	102.648	39	61.84	53.5876
67.5	114.546		36.72	71	153
29	64.1368		24.5	53.738	71.28

Tumor volumes (mm^3, R1-AD1 xenografts)

R1AD1-WT OE W3	R1AD1-WT OE W4	R1AD1-WT OE W5	R1AD1-DSM OE W3	R1AD1-DSM OE W4	R1AD1-DSM OE W5
70.2	169	306	53.2	129.71	187.7808
50.84	96.8	278.57	38.64	93.79	158.5488
79.12	162	262.159	29.5	70.47	122.6064
55.37	103.5	227.2	26.64	55.279	149.5
49.7	110.424	172.996	37.2	73.55	154
40.3	65.6	157.2			

Tumor weight (g, R1-AD1 xenografts)

Control	KD	WT OE	DSM OE
0.6861	0.51	1.8	0.843
0.5282	0.642	1.28	0.652
1	0.129	1.12	0.5
0.4	0.832	1	0.676
	0.21	0.72	0.43
	0.624	0.846	

Fig. S11c

Relative Daxx mRNA level (RT-qPCR)	
E0771-CTL	E0771-shDaxx
0.976	0.368
0.914	0.197
1.11	0.178

Tumor weight (g)	
E0771 CTL	E0771 shDaxx1
4.4	1.72
3.6	1.41
3.5	1.04
3.52	0.12

Fig. S11d

Tumor weight (g)	
shControl	shDaxx1
1.1	0.2
0.9	0.2
1.2	0.21
1	0.18
1	

Relative Daxx mRNA level (RT-qPCR)	
TRAMP-C2-CTL	TRAMP-C2-shDaxx
1.065	0.55
0.998	0.584
0.937	0.943

Fig. S12b

SIM2-SUMO1 FP measurement		
Log [SUMO-1] M	mP	SD
	15.05	1.061
-7.022	15.55	2.899
-6.721	16.3	0.141
-6.409	18.3	0.99
-6.108	18.6	0.707
-5.807	20.15	0.495
-5.504	25.35	2.051
-5.204	32.5	0.566
-4.903	45.4	0.424
-4.602	61.6	2.687
-4.301	76.4	0.283

Fig. S12c

Relative lipogenesis			
Control		DAXX OE	
None	SIM2	None	SIM2
1203.136	1066.147	1753.184	1515.058
1456.723	1150.897	1690.233	1347.464
1423.287	1095.49	1696.721	1475.742

Fig. S12d

Normalized radioactivity (MDA-MB-468 cells)	
None	SIM2
49644.733	42100.334
42383.2283	37068.6947
46032.8849	38108.7296

Normalized radioactivity (Hs578t cells)	
None	SIM2
20457	16172
21293.33	17374
22375	15367

Fig. S12e

Normalized radioactivity (R1-AD1 cells)	
None	SIM2
34825.02	28021.54
31428.68	28882.63
31884.91	30484.27

Normalized radioactivity (R1-D567 cells)	
None	SIM2
30074.37	23370.9
30448.92	21657.71
26789.72	24131.47

Fig. S12f

Normalized radioactivity (HCT116)	
None	SIM2
4180.963	3131.263
4371.048	3319.903
3755.43	3143.836

Fig. S12g

Normalized radioactivity (4T1)	
None	SIM2
504.6735	423.7836
791.003	428.9999
509.4472	393.466

Fig. S12h

Normalized radioactivity (4T1)	
None	SIM2
2410.668	2124.103
2328.97	2193.627
2663.618	1817.247

Fig. S12i

Control	SIM2 (10 μM)
103.293	87.71
96.624	83.683
100.083	86.809

Fig. S12j

MDA-MB-231 Control cells					
SREBF2 (CTL)	SREBF2 (SIM2)	FASN (CTL)	FASN (SIM2)	SCD (CTL)	SCD (SIM2)
1.017	0.848	1.203	0.718	1.144	0.623
0.951	0.637	1.092	0.634	1.031	0.513
1.032	1.309	0.704	0.861	0.825	0.989

MDA-MB-231 WT DAXX OE					
SREBF2 (CTL)	SREBF2 (SIM2)	FASN (CTL)	FASN (SIM2)	SCD (CTL)	SCD (SIM2)
0.896	0.447	1.086	0.486	1.023	0.853
0.911	0.498	1.041	0.406	1.081	0.723
1.193	0.424	0.872	0.333	0.896	0.779
