# Supplementary material for: DAXX drives de novo lipogenesis and contributes to tumorigenesis
Source: Nat Commun. 2023 Apr 12;14:1927. doi: 10.1038/s41467-023-37501-0 (PMC10097704; doi:10.1038/s41467-023-37501-0)
Supplement: Supplementary file 3 — Reporting Summary [file 41467_2023_37501_MOESM3_ESM.pdf]

nature portfolio

Corresponding author(s): Daiqing Liao

Last updated by author(s): 2023/03/15

Reporting Summary

Nature Portfolio wishes to improve the reproducibility of the work that we publish. This form provides structure for consistency and transparency in reporting. For further information on Nature Portfolio policies, see our Editorial Policies and the Editorial Policy Checklist.

Statistics

For all statistical analyses, confirm that the following items are present in the figure legend, table legend, main text, or Methods section.

n/a	Confirmed	
☐	☒	The exact sample size (*n*) for each experimental group/condition, given as a discrete number and unit of measurement
☐	☒	A statement on whether measurements were taken from distinct samples or whether the same sample was measured repeatedly
☐	☒	The statistical test(s) used AND whether they are one- or two-sided<br>*Only common tests should be described solely by name; describe more complex techniques in the Methods section.*
☒	☐	A description of all covariates tested
☐	☒	A description of any assumptions or corrections, such as tests of normality and adjustment for multiple comparisons
☐	☒	A full description of the statistical parameters including central tendency (e.g. means) or other basic estimates (e.g. regression coefficient) AND variation (e.g. standard deviation) or associated estimates of uncertainty (e.g. confidence intervals)
☐	☒	For null hypothesis testing, the test statistic (e.g. *F*, *t*, *r*) with confidence intervals, effect sizes, degrees of freedom and *P* value noted<br>*Give P values as exact values whenever suitable.*
☒	☐	For Bayesian analysis, information on the choice of priors and Markov chain Monte Carlo settings
☒	☐	For hierarchical and complex designs, identification of the appropriate level for tests and full reporting of outcomes
☒	☐	Estimates of effect sizes (e.g. Cohen's *d*, Pearson's *r*), indicating how they were calculated

Our web collection on statistics for biologists contains articles on many of the points above.

Software and code

Policy information about availability of computer code

Data collection

WB images were either acquired by Fuji film or GE Amersham Imager 680. qPCR data were acquired with Applied Biosystems 7500 Fast Real-Time PCR system. LC-MS data were collected using Dionex Ultimate 3000 UHPLC system coupled to a Q Exactive™ hybrid quadrupole-orbitrap mass spectrometer. The radioactivity data were determined with a liquid scintillation counter (Beckman LS 5000TD). Confocal images were acquired using Zeiss Laser Scanning Confocal Microscope (LSM) 800 with Zen blue software under identical setting and by using Plan-Apochromat 63x/1.40 oil DIC M27 objective. Fluorescence images were acquired with a Zeiss AxioPhot equipped with an Exi Blue camera (Qimaging).
Microarray data were acquired with the Affymetrix GeneChip Human Transcriptome Array 2.0. RNA-seq and ChIP-seq data were acquired using an Illumina Hi-Seq 2500 sequencer.

Data analysis

Data were analyzed using Microsoft Excel for Mac, Prism 9 for macOS, ImageJ 1.53a

Ingenuity Pathway Analysis (Version: 84978992 and Build: ing_jade)

Microarray analysis: RMA algorithm/R package, Affymetrix expression and transcriptome console software from ThermoFisher Scientific

RNA-seq:
HISAT2 (version 2.2.1-3n by Daehwan Kim (infphilo@gmail.com, www.ccb.jhu.edu/people/infphilo)
StringTie, version v1.3.4
Ballgown, version v2.12.0
DEG analysis: R packages: edgeR version 3.38.4

ChIP-seq: Bowtie2, SeqMINER, HOMER

For manuscripts utilizing custom algorithms or software that are central to the research but not yet described in published literature, software must be made available to editors and reviewers. We strongly encourage code deposition in a community repository (e.g. GitHub). See the Nature Portfolio guidelines for submitting code & software for further information.

Data

Policy information about availability of data

All manuscripts must include a data availability statement. This statement should provide the following information, where applicable:

- Accession codes, unique identifiers, or web links for publicly available datasets
- A description of any restrictions on data availability
- For clinical datasets or third party data, please ensure that the statement adheres to our policy

The microarray and RNA-seq data generated in this study have been deposited at NCBI under accession codes GSE190596, GSE223583, and GSE192420. The ChIP-seq data generated in this study has been deposited at NCBI under accession code GSE190783. Source data are provided with this paper.

Human research participants

Policy information about studies involving human research participants and Sex and Gender in Research.

Reporting on sex and gender	n/a
Population characteristics	n/a
Recruitment	n/a
Ethics oversight	n/a

Note that full information on the approval of the study protocol must also be provided in the manuscript.

Field-specific reporting

Please select the one below that is the best fit for your research. If you are not sure, read the appropriate sections before making your selection.

☒ Life sciences ☐ Behavioural & social sciences ☐ Ecological, evolutionary & environmental sciences

For a reference copy of the document with all sections, see nature.com/documents/nr-reporting-summary-flat.pdf

Life sciences study design

All studies must disclose on these points even when the disclosure is negative.

Sample size	No statistical analysis has been used to predetermine sample sizes in both in vivo or in vitro experiments. The sample sizes for in vitro and in vivo experiments were based on our previous studies (e.g., PMID: 23625935, PMID: 34199844) as well as relevant literature.
Data exclusions	No data were excluded for analysis
Replication	All experiments were performed at least in two biological duplicates with similar results. For in vivo experiments, individual tumor-bearing mouse served as replicates as specified in relevant figure legends.
Randomization	Tumor-bearing mice were randomized so that the initial tumor volume was equal in all treatment cohorts.
Blinding	Researchers were not blinded to allocation of animals during experiments and outcome assessments. They implanted tumor cells, measured tumor sizes, injected drugs, processed and stored samples, and analyzed data, which is incompatible with blinding.

Reporting for specific materials, systems and methods

We require information from authors about some types of materials, experimental systems and methods used in many studies. Here, indicate whether each material, system or method listed is relevant to your study. If you are not sure if a list item applies to your research, read the appropriate section before selecting a response.

Materials & experimental systems

n/a	Involved in the study	
☐	☒	Antibodies
☐	☒	Eukaryotic cell lines
☒	☐	Palaeontology and archaeology
☐	☒	Animals and other organisms
☒	☐	Clinical data
☒	☐	Dual use research of concern

Methods

n/a	Involved in the study	
☐	☒	ChIP-seq
☒	☐	Flow cytometry
☒	☐	MRI-based neuroimaging

Antibodies

Antibodies used

DAXX (for IB), Bethyl laboratories, A301-352A, lot 1, 1:20,000 dilution
DAXX (for IB), Bethyl laboratories, A301-353A, lot 1, 1:20,000 dilution
DAXX (for IP, IB, IF PLA, and ChIP), The Developmental Studies Hybridoma Bank, PCRP-DAXX-5G11, Hybridoma supernatant. 1:100 dilution for IB, 1:5 for IF, and 1:2 for PLA
DAXX (for IP and IB) GenScript, rabbit polyclonal, custom-made antibody for this study 1:10,000 dilution for IB
FASN (for IB and IF) ProteinTech, 10624-2-AP, lot 00039886, 1:20,000 dilution for IB, and, 1:700 for IF
FASN, Santa Cruz, SC-55580 (A-5), lot B2316, 1:20,000 dilution (IB)
ACC1, Cell Signaling Technology, 3676 (C83B10), lot 8, 1:20,000 dilution (IB)
ACLY, Cell Signaling Technology, 13390 (D1X6P), lot 1, 1:10,000 dilution (IB)
ACSS2, Cell Signaling Technology, 3658 (D19C6), lot 2, 1:20,000 dilution (IB)
SREBP2, Cayman Chemical, 10007663, lot 0480271-1, 1:10,000 dilution for IB, 1:300 for IF and 1:100 for PLA
SREBP2, BD Biosciences, clone IgG-1C6, 557037, lot 5166992, 1:1,000 dilution for IB
SREBP1, Santa Cruz, SC-13551, lot C2416, 1:500 dilution (IB)
SREBP1, ProteinTech, 14088-1-AP, lot 00048393, 1:3,000 dilution for IB, 1:300 for IF and 1:100 for PLA
FLAG, Cell Signaling Technology, 14793, lot 4, 1:10,000 (IB)
FLAG (IB, IP and ChIP), Millipore-Sigma, F1804, various lots, 1:1,000 dilution (IB), 10 μg for IP and ChIP
GFP, Cell Signaling Technology, 2956, lot 4, 1:3,000 dilution (IB)
PCNA, Epitomics, 2714-1, clone EPR3821, lot YG-03-25-14C, 1:20,000 dilution (IB)
alpha-Tubulin, Millipore-Sigma, clone B-5-1-2, T5168, 1:50,000 dilution (IB)
HSP60, BD Transduction Laboratories, H99020, 1:50,000 dilution (IB)
Rabbit IgG HRP-linked antibody, Cell Signaling Technology, 7074, various lots, 1:10,000 dilution (IB)
Mouse IgG HRP-linked antibody, Cell Signaling Technology, 7076, various lots, 1:10,000 dilution (IB)
Normal mouse IgG (for IP/ChIP control) Santa Cruz, SC-2025, lot D1216, diluted to 2 μg per experiment

Validation

All antibodies were validated by vendors and validation data are available at vendors' website:

DAXX (for IB), Bethyl laboratories, A301-352A. This antibody is no longer available from the vendor. This antibody has been validated by shRNA-mediated DAXX knockdown and cDNA overexpression in this study

DAXX (for IB), Bethyl laboratories, A301-353A: https://www.fortislife.com/products/primary-antibodies/rabbit-anti-daxx-antibody/BETHYL-A301-353

DAXX (for IP, IB, IF PLA, and ChIP), The Developmental Studies Hybridoma Bank, PCRP-DAXX-5G11, Hybridoma supernatant. 1:100 dilution for IB, 1:5 for IF, and 1:2 for PLA: This antibody has been validated by shRNA-mediated DAXX knockdown and cDNA overexpression in this study

DAXX (for IP and IB) GenScript, rabbit polyclonal, custom-made antibody for this study: This antibody has been validated by shRNA-mediated DAXX knockdown and cDNA overexpression in this study

FASN (for IB and IF) ProteinTech, 10624-2-AP: https://www.ptglab.com/products/FASN-Antibody-10624-2-AP.htm

FASN (IB), Santa Cruz, SC-55580 (A-5): https://www.scbt.com/p/fatty-acid-synthase-antibody-a-5

ACC1, Cell Signaling Technology, 3676 (C83B10): https://www.cellsignal.com/products/primary-antibodies/acetyl-coa-carboxylase-c83b10-rabbit-mab/3676

ACLY, Cell Signaling Technology, 13390 (D1X6P): https://www.cellsignal.com/products/primary-antibodies/atp-citrate-lyase-d1x6p-rabbit-mab/13390?_=1676934724070&Ntt=13390&tahead=true

ACSS2, Cell Signaling Technology, 3658 (D19C6): https://www.cellsignal.com/products/primary-antibodies/acecs1-d19c6-rabbit-mab/3658

SREBP2 (IB and PLA), Cayman Chemical, 10007663: https://www.citeab.com/antibodies/2864481-10007663-srebp-2-polyclonal-antibody?des=fab8abf3a253a277. This antibody has also been validated by shRNA-mediated SREBP2 knockdown and cDNA overexpression in this study.

SREBP2 (IB), BD Biosciences, clone IgG-1C6, 557037: https://www.citeab.com/antibodies/2410727-557037-bd-pharmingen-purified-

mouse-anti-srebp-2. This antibody has also been validated by shRNA-mediated SREBP2 knockdown and cDNA overexpression in this study.

SREBP1 (IB), Santa Cruz, SC-13551, clone 2A4: https://www.scbt.com/p/srebp-1-antibody-2a4

SREBP1 (IB, IF and PLA), ProteinTech, 14088-1-AP: https://www.ptglab.com/products/SREBF1-Antibody-14088-1-AP.htm

FLAG (IB), Cell Signaling Technology, 14793: https://www.cellsignal.com/products/primary-antibodies/dykddddk-tag-d6w5b-rabbit-mab-binds-to-same-epitope-as-sigma-s-anti-flag-m2-antibody/14793?_=1676934938813&Ntt=14793&tahead=true

FLAG (IB, IP and ChIP), Millipore-Sigma, F1804, clone M2: https://www.sigmaaldrich.com/US/en/product/sigma/f1804

GFP (IB), Cell Signaling Technology, 2956: https://www.cellsignal.com/products/primary-antibodies/gfp-d5-1-rabbit-mab/2956

PCNA (IB), Epitomics, 2714-1, clone EPR3821: https://www.abcam.com/pcna-antibody-epr3821-ab92552.html

alpha-Tubulin (IB), Millipore-Sigma, clone B-5-1-2, T5168: https://www.sigmaaldrich.com/US/en/product/sigma/t5168

Rabbit IgG HRP-linked antibody (IB), Cell Signaling Technology, 7074: https://www.cellsignal.com/products/secondary-antibodies/anti-rabbit-igg-hrp-linked-antibody/7074

Mouse IgG HRP-linked antibody (IB), Cell Signaling Technology, 7076: https://www.cellsignal.com/products/secondary-antibodies/anti-mouse-igg-hrp-linked-antibody/7076

Normal mouse IgG (for IP/ChIP control) Santa Cruz, SC-2025: https://www.scbt.com/p/normal-mouse-igg?requestFrom=search

Eukaryotic cell lines

Policy information about cell lines and Sex and Gender in Research

Cell line source(s)	Human cell lines (MDA-MB-231, MDA-MB-468, Hs578t, MCF7, T47D, HCT116, PC-3, and 293T) were obtained from ATCC (Manassas, VA). R1-AD1 and R1-D567 were provided by Dr. Scott Dehm, University of Minnesota.<br><br>The mouse cancer cell lines 4T1, CT26.CL25, and TRAMP-C2 were from ATCC. The mouse BC cell line E0771 was from CH3 BioSystems (Amherst, NY).
Authentication	Human cell lines (MDA-MB-231, MDA-MB-468, Hs578t, MCF7, T47D, PC-3, HCT116) and mouse 4T1 cell line were authenticated by STR profiling at Genetica DNA Laboratories (Burlington, NC). The other cell lines (293T, CT26.CL25, TRAMP-C2, E0771, R1-AD1, and R1-D567) were recently acquired from vendors or academic labs and were not subjected to further authentication.
Mycoplasma contamination	Cell cultures were routinely examined for potential mycoplasma contamination by DAPI staining and fluorescence microscopy. Possibly contaminated cell lines were treated with ciprofloxacin (10 µg/ml) and reexamined to confirm the absence of mycoplasma contamination. All cell cultures were negative of mycoplasma contamination.
Commonly misidentified lines<br>(See ICLAC register)	No commonly misidentified cell lines were used.

Animals and other research organisms

Policy information about studies involving animals; ARRIVE guidelines recommended for reporting animal research, and Sex and Gender in Research

Laboratory animals	All mice were housed in pathogen-free facilities (SPF), under standard conditions, in ventilated cages with enrichment, at 72 degrees Fahrenheit, 60% humidity, 12 hour light/ 12 hour dark cycles, and standard water and diet in the University of Florida animal facilities. Female and male mice (6-12 week old) of the strains of NSG (NOD.Cg-Prkdc-scid-Il2rg-tm1Wjl/SzJ) , BALB/c and CL57BL/6 were used for this study.
Wild animals	No wild animals were used.
Reporting on sex	Female or male mice were used for breast or prostate cancer models, respectively. Both male and female mice for other cancer models
Field-collected samples	No field-collected samples were involved.
Ethics oversight	University of Florida IACUC

Note that full information on the approval of the study protocol must also be provided in the manuscript.

ChIP-seq

Data deposition

☒ Confirm that both raw and final processed data have been deposited in a public database such as GEO.

☒ Confirm that you have deposited or provided access to graph files (e.g. BED files) for the called peaks.

Data access links<br>*May remain private before publication.*	https://www.ncbi.nlm.nih.gov/geo/query/acc.cgi?acc=GSE190783
Files in database submission	shControl_IM7_S26_L004_R1_001.fastq.gz fastq for Chipseq of DAXX, shControl cell<br>WT DAXX OE_IM8_S27_L004_R1_001.fastq.gz fastq fastq for Chipseq of DAXX, DAXX WT OE cells<br>DAXX DSM OE_IM9_S28_L004_R1_001.fastq.gz fastq fastq for Chipseq of DAXX, DSM OE cells
Genome browser session<br>(e.g. UCSC)	https://genome.ucsc.edu/cgi-bin/hgTracks?<br>db=hg38&lastVirtModeType=default&lastVirtModeExtraState=&virtModeType=default&virtMode=0&nonVirtPosition=&posit<br>ion=chr2%3A25160915%2D25168903&hgsid=1558821113_6vJiDt1ZoSV8AazAOxke840wOAc2

Methodology

Replicates	Samples were run using one replicate.
Sequencing depth	IM7_S26_L004_R1_001.fastq.gz: 41,610,648<br>IM8_S27_L004_R1_001.fastq.gz: 36,963,873<br>IM9_S29_L004_R1_001.fastq.gz: 34,897,893
Antibodies	PCRP-DAXX-5G11, Hybridoma supernatant
Peak calling parameters	Default parameters were used
Data quality	Default Homer setting was used, including<br>FDR rate threshold = 0.001000<br>Fold over local region required = 4.00<br>Poisson p-value over local region required = 1.00e-04<br>Fold over local region required = 4.00<br>Poisson p-value over local region required = 1.00e-04<br>Putative peaks filtered for being too clonal = 3
Software	Bowtie, HOMER
